# Structural insights of human mitofusin-2 into mitochondrial fusion and CMT2A onset

**DOI:** 10.1038/s41467-019-12912-0

**Published:** 2019-10-29

**Authors:** Yu-Jie Li, Yu-Lu Cao, Jian-Xiong Feng, Yuanbo Qi, Shuxia Meng, Jie-Feng Yang, Ya-Ting Zhong, Sisi Kang, Xiaoxue Chen, Lan Lan, Li Luo, Bing Yu, Shoudeng Chen, David C. Chan, Junjie Hu, Song Gao

**Affiliations:** 1 State Key Laboratory of Oncology in South China, Collaborative Innovation Center for Cancer Medicine, Sun Yat-sen University Cancer Center, 510060 Guangzhou, China; 2 Department of Genetics and Cell Biology, College of Life Sciences, Nankai University, 300071 Tianjin, China; 3 Division of Biology and Biological Engineering, California Institute of Technology, Pasadena, CA USA; 4 Department of Experimental Medicine, Guangdong Provincial Key Laboratory of Biomedical Imaging, The Fifth affiliated Hospital, Sun Yat-sen University, 519000 Zhuhai, China; 5 National Laboratory of Biomacromolecules, CAS Center for Excellence in Biomacromolecules, Institute of Biophysics, Chinese Academy of Sciences, 100101 Beijing, China; 6 University of Chinese Academy of Sciences, 100101 Beijing, China; 7 Guangzhou Regenerative Medicine and Health Guangdong Laboratory, 510530 Guangzhou, China

**Keywords:** Enzyme mechanisms, Mitochondria, X-ray crystallography, Neurodegenerative diseases

## Abstract

Mitofusin-2 (MFN2) is a dynamin-like GTPase that plays a central role in regulating mitochondrial fusion and cell metabolism. Mutations in *MFN2* cause the neurodegenerative disease Charcot-Marie-Tooth type 2A (CMT2A). The molecular basis underlying the physiological and pathological relevance of MFN2 is unclear. Here, we present crystal structures of truncated human MFN2 in different nucleotide-loading states. Unlike other dynamin superfamily members including MFN1, MFN2 forms sustained dimers even after GTP hydrolysis via the GTPase domain (G) interface, which accounts for its high membrane-tethering efficiency. The biochemical discrepancy between human MFN2 and MFN1 largely derives from a primate-only single amino acid variance. MFN2 and MFN1 can form heterodimers via the G interface in a nucleotide-dependent manner. CMT2A-related mutations, mapping to different functional zones of MFN2, lead to changes in GTP hydrolysis and homo/hetero-association ability. Our study provides fundamental insight into how mitofusins mediate mitochondrial fusion and the ways their disruptions cause disease.

## Abstract (web summary)

Mitofusin-2 (MFN2) is a dynamin-like GTPase that plays a central role in regulating mitochondrial fusion and cell metabolism. Here, authors report crystal structures of truncated human MFN2 in different nucleotide-loading states and show that MFN2 forms sustained dimers even after GTP hydrolysis.

## Introduction

Mitochondria are double-membrane organelles with varying shapes influenced by metabolic conditions, developmental stage, and environmental stimuli^1–3^. Their highly dynamic morphology is realized through regulated and balanced fusion and fission processes^4,5^. Fusion is important for the health and physiological functions of mitochondria, including complementation of damaged mitochondrial DNAs and maintenance of membrane potential^5,6^. Outer mitochondrial membrane (OMM) fusion is catalyzed by the dynamin-like GTPase called mitofusin (MFN)^7,8^. Mammals have two broadly expressed and essential mitofusins, namely MFN1 and MFN2. The two mitofusins share ~80% sequence similarity, and display a certain grade of functional redundancy in mice^9,10^.

Despite the similarities, MFN1 and MFN2 are also reported in an in vitro study to play distinct roles in mediating mitochondrial fusion via GTPase activity^11^. Compared with MFN1, MFN2 seems more versatile, and is frequently related to human diseases. MFN2 is proposed to regulate ER–mitochondria juxtaposition^12,13^. During the mitophagy process, MFN2 is reported to be phosphorylated by mitochondrial PTEN-induced putative kinase 1, and then recognized by Parkin for culling damaged mitochondria^14–16^. Functions of MFN2 have also been suggested in several other cellular processes including ER stress, axonal transport, and cell cycle progression^17–21^. Mutations in *MFN2*, but not *MFN1*, cause most cases of Charcot–Marie–Tooth type 2A (CMT2A) disease, an incurable inherited neuromuscular disorder^22^. Emerging evidence suggests that abnormal expression of MFN2 is also associated to the onset of many other human diseases including Alzheimer’s disease, Parkinson’s disease, cardiomyopathy, diabetes, and cancer^23–30^. However, the structural basis for the functional diversity and pathophysiological relevance of human MFN2 remains unclear.

Our recent structural studies of truncated MFN1 revealed a typical dynamin-like domain organization of mitofusins, and proposed a model for mitochondrial tethering involving intermolecular *(trans*) association of G domains and subsequent GTP hydrolysis-induced power stroke^31–33^. Although the overall architecture of MFN2 is expected to be similar as that of MFN1, the current information is still insufficient to explain the functional discrepancy of the two mitofusins. Thus, a detailed structural analysis of MFN2 is critical to pinpoint the crucial differences between the two mitofusins on the molecular level. In addition, MFN2 and MFN1 are shown to physically contact each other in cells, and this hetero-association may play an important role in mediating mitochondrial fusion and ER–mitochondria tethering^9,13,34^, but the molecular basis of their interaction has yet to be elucidated.

In this study, we address these issues by presenting crystal structures of truncated human MFN2 in different stages of the GTP turnover cycle. These structures explain how MFN2 uses a primate-only single amino acid variance and a tighter G interface, as compared with human MFN1, to allow for enduring GTPase (G) domain association even after GTP hydrolysis, a feature that is unique in the dynamin superfamily and ensures efficient membrane tethering. We also found that MFN2 is able to bind MFN1 via the G interface and performs domain rearrangement in a nucleotide-dependent manner. The structures of MFN2 enable us to visualize most of the CMT2A-related mutations in a three-dimensional landscape, and explain the unexpected diverse biochemical phenotypes of these mutants and mechanisms of MFN2-related CMT2A onset. Overall, out study greatly contributes to the understanding of MFN2-mediated cellular activities and the ways its disruption cause disease.

## Results

### Overall structure of truncated MFN2

**Fig. 1:**
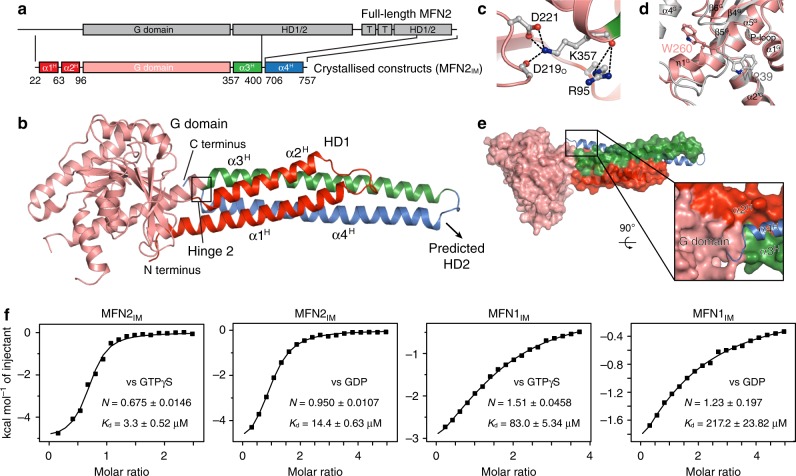
Overall structure of truncated MFN2_IM_ **a** Schematic representation showing the organization of MFN2_IM_ based on full-length MFN2. G domain, GTPase domain; HD1/2, helical domain 1/2; T, transmembrane region. Elements for MFN2_IM_ are assigned according to the structure. Borders of each element are indicated by residue numbers. **b** Structure of nucleotide-free MFN2_IM_. α-helices of HD1 are differentially colored to specify their distribution on the primary structure as in **a**. **c** Details of hinge 2 of MFN2_IM_ in nucleotide-free state. **d** Structural comparison between Trp260 in MFN2_IM_ (pink) and Trp239 in MFN1_IM_ (gray) in nucleotide-free states. **e** The C terminus of ɑ4^H^ physically contacts the G domain. Nucleotide-free MFN2_IM_ is illustrated here. **f** Binding affinities (dissociation constant, *K*_d_) to guanine nucleotides for nucleotide-free MFN2_IM_ and MFN1_IM_ measured by isothermal titration calorimetry (ITC)

We generated a modified human MFN2 construct with deletions of residues 1–21 and 401–705 (MFN2_IM_). When purified from *Escherichia* *coli*, MFN2_IM_ was loaded with GTP or GDP, which could be removed by an optimized purification protocol (Supplementary Fig. 1a, b). We determined crystal structures of MFN2_IM_ in the nucleotide-free and GDP-bound forms at 2.8 and 2.0 Å, respectively (Fig. 1a, b, Table 1). MFN2_IM_ contains a G domain and a helical domain 1 (HD1). HD1 is connected to the G domain via the so-called hinge 2, namely Arg95 at the C-terminal end of α2^H^, and Lys357 between α5^G^ and α3^H^ (Fig. 1c). The G domain consists of a central nine-stranded β-sheet (β1^G^–β6^G^ and β1′^G^–β3′^G^) surrounded by seven ɑ-helices (ɑ1^G^–ɑ5^G^, ɑ1′^G^, and ɑ2′^G^) and two 3_10_-helices (η1^G^ and η2^G^), of which β2′^G^ and η1^G^ are not observed for MFN1_IM_ (Supplementary Fig. 2a). Unlike MFN1, MFN2_IM_ has a disordered switch I in the nucleotide-free state, and the Trp260 does not occupy the nucleotide-binding pocket (Fig. 1d). The four-helical-bundle HD1 is stabilized by a massive hydrophobic network (Supplementary Fig. 2b). ɑ4^H^, which comprises large portion of the conventional HR2, is substantially involved in this network. The C-terminal tip of ɑ4^H^ physically contacts the G domain (Fig. 1e, Supplementary Fig. 2c). With more residues preserved than in the crystallized MFN1_IM_ construct^32^, ɑ3^H^ and ɑ4^H^ are extended in MFN2_IM_ (Fig. 1b, Supplementary Fig. 2d). A recently predicted MFN2 model proposed intramolecular interactions for Met376-Leu727, and for His380-Asp725 between ɑ3^H^ and ɑ4^H^ ^35^. In our crystal structures, however, Met376 is surrounded by Phe56, Leu57, and Leu734 from the neighboring helices, and separated from Leu727. His380 and Asp725 are both on the surface, but their side chains are not close enough to form a salt bridge (Supplementary Fig. 2e).

**Table 1:** Crystallographic data collection and refinement

State	MFN2_IM_	MFN2_IM_	MFN2_IM_(T111D)
	apo	GDP	apo
Data collection			
Space group	P12_1_1	P4_1_2_1_2	P12_1_1
Cell dimensions			
*a*, *b*, *c* (Å)	84.5, 128.4, 91.3	49.6, 49.6, 388.1	44.4, 126.6, 78
α, β, γ (°)	90, 106.3, 90	90	90, 102.5, 90
Wavelength (Å)	0.97915	0.977760	0.97914
Resolution (Å)	48.7–2.8 (2.98–2.81)	49.6–2.0 (2.12–2.00)	126.6–2.1 (2.14–2.09)
* R*_sym_^a^	0.106 (0.490)	0.058 (0.475)	0.070 (0.573)
* I*/σ(*I*)	10.42 (3.05)	18.31 (3.22)	10.6 (2.2)
Completeness (%)	98.0 (94.5)	99.3 (98.2)	98.8 (99.5)
Redundancy	3.3 (3.3)	6.2 (5.9)	4.4 (4.3)
Refinement			
Resolution (Å)	36.3–2.8	48.1–2.0	31.6–2.1
No. reflections	44910	34449	50316
* R*_work_/*R*_free_	0.207/0.283	0.176/0.212	0.176/0.229
No. atoms			
Protein	13347	3424	6824
Ligand/ion	7	65	11
Water	132	171	268
*B*-factors			
Protein	52.3	43.9	45.5
Ligand/ion	71.2	51.0	61.2
Water	39.6	46.2	48.9
R.m.s. deviations			
Bond lengths (Å)	0.009	0.007	0.008
Bond angles (°)	1.184	0.903	0.943

^a^Numbers in parentheses represent values from the highest resolution shell

We tested the association of nucleotide-free MFN2_IM_ and MFN1_IM_ with different guanine nucleotides by isothermal titration calorimetry (ITC). MFN2_IM_ showed much higher affinity to GTPγS and GDP compared with MFN1_IM_, and could efficiently bind to GMPPNP (Fig. 1f, Supplementary Fig. 2f). The stronger nucleotide association of MFN2_IM_ may be partly explained by the aforementioned conformational variance to MFN1_IM_: the disordered switch I and outwardly oriented Trp260 leave an empty pocket that facilitates nucleotide docking (Fig. 1d).

Thr111 in the P-loop of MFN2 has been reported to be a phosphorylation site^16^. To investigate how Thr111 phosphorylation affects the function of MFN2, we solved the crystal structure of a phosphorylation mimic MFN2_IM_(T111D) (Supplementary Fig. 3a). According to the structure, the side chain of Asp111 is not fully exposed, and its phosphorylation may require a substantial local conformational rearrangement. Phosphorylated Thr111, as reflected by the Asp111 in the crystal structure, blocks the binding groove for GTP in the P-loop, and the negative charge is also disfavored for the phosphate groups of the nucleotide (Supplementary Fig. 3b). Subsequent ITC analysis confirmed that MFN2_IM_(T111D) has no binding affinity to guanine nucleotides (Supplementary Fig. 3c). Therefore, the putative phosphorylation at Thr111 is likely to directly inactivate MFN2 in mediating mitochondrial fusion. Intriguingly, we observed calcium ions (Ca^2+^) associated with the HD1 of both nucleotide-free wild-type MFN2_IM_ and MFN2_IM_(T111D), and one of them is involved in intermolecular contact (Supplementary Fig. 3d). Addition of Ca^2+^ did not alter the biochemical features of wild-type MFN2_IM_ or MFN2_IM_(T111D) (Supplementary Fig. 3e, f).

### MFN2_IM_ is an extremely weak GTPase

**Fig. 2:**
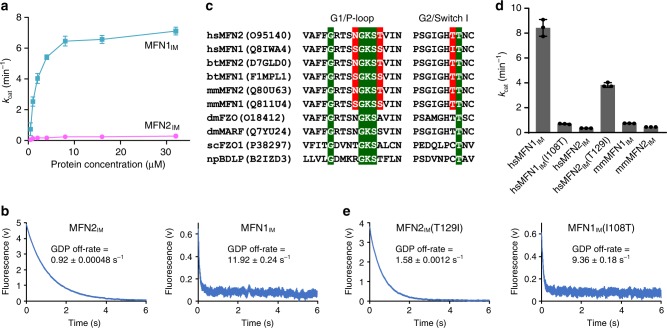
MFN2 is a weak GTPase. **a** GTP turnover rates of MFN2_IM_ and MFN1_IM_ were measured at seven different protein concentrations. Error bars indicate s.d. (*n* = 3). **b** GDP off-rates of MFN2_IM_ and MFN1_IM_. A representative trace from three independent experiments is shown for each sample. **c** Sequence alignment of the P-loop and switch I of mitofusins from various species. Conventional motifs/residues are highlighted in green. Residues that differ between human MFN1 and MFN2 and may be responsible for GTP binding and hydrolysis are highlighted in red. hs *Homo sapiens,* bt *Bos taurus,* mm *Mus musculus,*; dm *Drosophila melanogaster,* sc *Saccharomyces cerevisiae,* np *Nostoc punctiforme*. **d** GTP turnover rates of wild-type human (hs) and mouse (mm)MFN1_IM_/MFN2_IM_, and indicated mutants. Error bars indicate s.d. (*n* = 3). **e** GDP off-rates of MFN2_IM_(T129I) and MFN1_IM_(I108T). Source data are provided as a Source Data file

Previous study showed that at low protein concentration (~0.06 μM), human MFN2 has eightfold lower GTPase activity than MFN1 in the presence of mild detergent^11^. We compared the GTP turnover rates between MFN2_IM_ and MFN1_IM_ at different protein concentrations. MFN1_IM_ showed stimulated GTP turnover, and the maximum *k*_cat_ exceeded 7 min^−1^ in the presence of 150 mM KCl. In contrast, the GTP turnover of MFN2_IM_ remained less than 0.4 min^−1^ regardless of increasing protein concentrations in the same condition (Fig. 2a). This huge difference in GTPase activity prompted us to check the GDP off-rates of MFN1_IM_ and MFN2_IM_ by fast kinetics analysis. In stopped-flow experiments, the GDP off-rate for MFN2_IM_ was slower than that of MFN1_IM_ by 13-fold (Fig. 2b). Thus, the weak GTPase activity of MFN2 can be partly explained by the less efficient nucleotide exchange, which is also in agreement with its higher binding affinity to guanine nucleotides (Fig. 1f)

We then investigated the reasons for these differences between the two homologs. Human MFN1 and MFN2 bear several different residues in the P-loop and switch I, namely MFN2-Asn107/MFN1-Ser86, MFN2-Thr111/MFN1-Ser90, and MFN2-Thr129/MFN1-Ile108 (Fig. 2c). We individually swapped corresponding residues between MFN1_IM_ and MFN2_IM_, and applied these mutants to GTP hydrolysis assays. MFN2_IM_(T129I) strongly promoted GTP turnover by ~11-fold, while MFN1_IM_(I108T) showed a 12-fold decrease (Fig. 2d). The other two swapping pairs, by contrast, did not significantly alter the GTPase activity of their wild-type counterparts (Supplementary Fig. 4a). Interestingly, rodent MFN1 and MFN2 both have a threonine at the corresponding position in switch I (Fig. 2c, Supplementary Fig. 4b). Mouse MFN1_IM_ and MFN2_IM_ showed weak GTPase hydrolysis similar to human MFN1_IM_(I108T) and human MFN2_IM_, respectively (Fig. 2d). These results suggest that the MFN2-Thr129/MFN1-Ile108 difference in switch I is a key determinant for the incongruent GTPase activity of human mitofusins.

We have previously reported that MFN1-Ile108 is involved in a hydrophobic cluster that stabilizes switch I in a nucleotide-occluding conformation^32^. The cluster is apparently disfavored by the Thr129 exchange in MFN2, which endows the observed flexibility to switch I in the nucleotide-free state (Supplementary Fig. 4c). This idea was supported by the fact that T129I swapping in MFN2_IM_ slightly reduced binding affinity to GTPγS and GDP, and increased GDP off-rate, whereas MFN1_IM_(I108T) led to opposite outcome (Fig. 2e, Supplementary Fig. 4d). This moderate influence, however, seems insufficient to explain the large variance in the GTPase activity between MFN1 and MFN2. In addition, we noticed that in all ITC results of MFN2_IM_, including MFN2_IM_(T129I), the *N* values (indicating numbers of binding site) are between 0.5 and 1, whereas for MFN1_IM_ samples the *N* values are all ranged from 1 to 1.5 (Fig. 1f, Supplementary Figs. 2f, 4d). For MFN1_IM_ constructs, the larger apparent *N* values might be derived from the conformation rearrangement of the Trp260 switch and switch I required for nucleotide loading^32^, but this does not explain the smaller *N* values of MFN2_IM_ in ITC experiments. Thus, there may be other factors that define the GTP turnover efficiency of human mitofusins, such as the dimerization of the GTPase domains.

### MFN2_IM_ remains dimerized after GTP hydrolysis

**Fig. 3:**
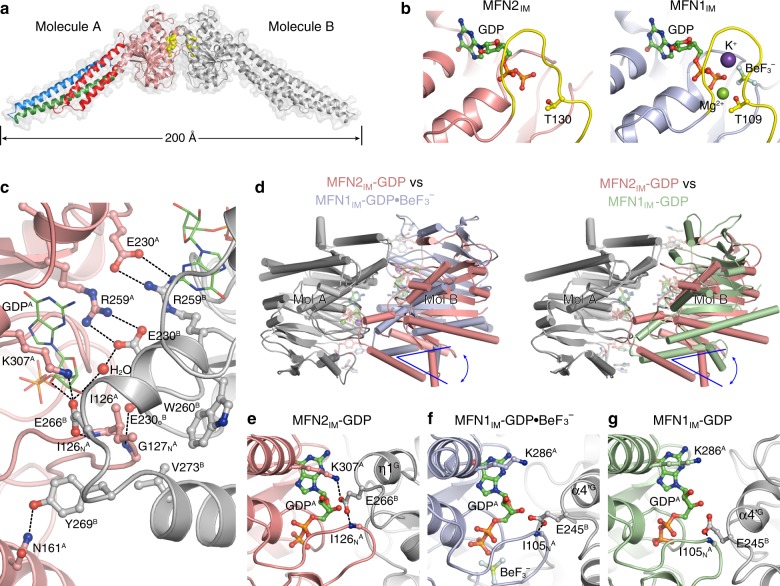
Dimerization of MFN2_IM_ via the G domain. **a** The MFN2_IM_ dimer in GDP-bound state, with transparent surface representation. Molecule A is colored as in Fig. 1b, molecule B is in gray. GDP is shown as yellow spheres. **b**, Switch I configuration of MFN2_IM_-GDP structure and MFN1_IM_ (Protein Data Bank code 5YEW) in the transition state. Switch I is colored yellow. The catalytic residues MFN2_IM_(Thr130) and MFN1_IM_(Thr109) are shown as ball-and-stick models. **c** Details of the G interface of MFN2_IM_. Only one side of the G interface is shown for other involved residues except for the central dual salt bridges. **d** Structural comparison of MFN2_IM_-GDP dimer with MFN1_IM_-${\mathrm{GDP}} \bullet {\mathrm{BeF}}_{3}^{-}$ dimer (PDB code 5YEW, left) and with MFN1_IM_-GDP dimer (PDB code 5GOM, right). The structures are superimposed for one polypeptide chain (Mol A, shown in gray). The positions of the other chain (Mol B) showed a clear difference in orientation between MFN2_IM_ (pink) and MFN1_IM_ structures (light blue or green). Comparison of the G interface of MFN2_IM_ in the GDP-bound state (**e**) between MFN1_IM_ in the transition state (**f** PDB code 5YEW) and in the GDP-bound state (**g** PDB code 5GOM). Note the MFN2-specific Glu266-Lys307 salt bridge and the tighter trans association

Dynamin superfamily members including MFN1, are known to form functional G domain-mediated dimers in a nucleotide-dependent manner^32,36–38^. As revealed by right-angle light scattering (RALS), MFN2_IM_ stayed monomeric in the nucleotide-free (apo) and GDP/GTPγS/GMPPNP-loading states, and formed stable dimers in the presence of GTP or ${\mathrm{GDP}} \bullet {\mathrm{BeF}}_{3}^{-}$ (Supplementary Fig. 5a). The MFN2_IM_-GTP dimers were sustained after further incubation while the GTP was slowly hydrolyzed to GDP (Supplementary Fig. 5b). For the MFN2_IM_ crystals that yielded the GDP-bound structure, the GDP was not added during purification or crystallization, but co-purified from the host *E*. *coli* cells (Supplementary Fig. 1a). Somewhat unexpectedly, this GDP-bound MFN2_IM_ forms a G domain-mediated dimer across the asymmetric units of the crystal lattice (Fig. 3a and Table 1). RALS analysis revealed that the majority of freshly purified MFN2_IM_ molecules were indeed in the dimeric form (Supplementary Fig. 5c). These observations indicate that MFN2_IM_ dimerizes during GTP hydrolysis, and the dimer remained associated after the reaction. In the crystal structure, two citrate ions were present between the associated G domains, and making contacts with both of them in a symmetrical manner (Supplementary Fig. 5d, e). In solution, however, citrate did not affect GTP hydrolysis, or induce dimerization of GDP-bound MFN2_IM_ (Supplementary Fig. 5f, g), suggesting that the MFN2_IM_-GDP dimer in the crystal structure is not dependent on citrate.

The area of the MFN2_IM_-GDP dimeric interface (G interface) excluding citrate ions is 1065 Å^2^, which exceeds the 984 Å^2^ interface of the MFN1_IM_-GDP dimer of the posttransition state^32^. By comparing the catalytic sites between our MFN2_IM_-GDP structure and MFN1_IM_ in the transition state (MFN1_IM_-${\mathrm{GDP}} \bullet {\mathrm{BeF}}_{3}^{-}$)^33^, we found that their switch I regions are very similar in overall conformations, as are the positions of the catalytic threonine (Fig. 3b). When the G domains are superimposed, the HD1 of MFN2_IM_ takes an open orientation similar to the MFN1_IM_-GDP dimer (Supplementary Fig. 6a). On the G interface of MFN2_IM_, the *trans* interactions include (i) the central dual salt bridges between Glu230 and Arg259; (ii) a hydrophobic cluster involving Ile126 on switch I and Trp260/Val273, and (iii) other hydrogen bonds and salt bridges (Fig. 3c). These contacts (e.g., the hydrophobic cluster) are more consistent with those of the MFN1_IM_-${\mathrm{GDP}} \bullet {\mathrm{BeF}}_{3}^{-}$ dimer. Thus, our MFN2_IM_-GDP dimer may represent a posttransition state right after GTP hydrolysis.

On the other hand, the component monomers of the MFN2_IM_ dimer stay in different relative positions as compared with the MFN1_IM_ dimer (Fig. 3d), which leads to some varied *trans* residue contacts in the G interface. For example, unlike in MFN1_IM_ in the transition state, Tyr269 of MFN2_IM_ only interacts with Asn161, but not with His168 (Fig. 3d and Supplementary Fig. 6b). More importantly, Glu266 forms an MFN2-specific *trans* salt bridge to Lys307, whose side chain is in parallel with the guanine group of the nucleotide (Fig. 3e). This association, together with the central salt bridge and η1^G^, tightly enwraps the nucleotide. For MFN1_IM_, corresponding residues are apparently relocated, resulting in a free space next to the nucleotide (Fig. 3f, g).

### Tight G interface ensures high tethering efficiency to MFN2

**Fig. 4:**
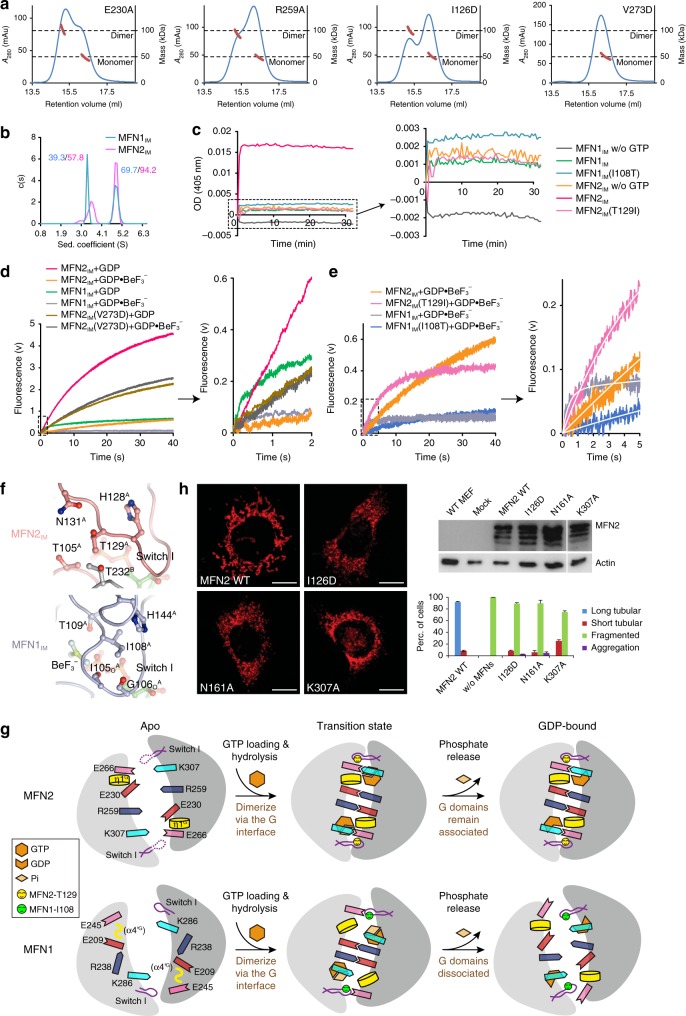
Characterization of MFN2_IM_ dimeric interface. **a** Dimerization property of the G interface mutants in the presence of ${\mathrm{GDP}} \bullet {\mathrm{BeF}}_{3}^{-}$ was assayed by analytical gel filtration coupled to RALS. Calculated molecular masses at the absorption peaks of 280 nm are plotted in red. mAU milli-absorption units. **b** AUC results of MFN2_IM_ and MFN1_IM_ (theoretical molecular mass 52.6 and 50.9 kDa, respectively) in the presence of ${\mathrm{GDP}} \bullet {\mathrm{BeF}}_{3}^{-}$. The estimated molecular masses determined by sedimentation velocity are given in kilodaltons (kDa) above the peaks. **c** Liposome tethering assay for wild-type MFN2_IM_-TM/MFN1_IM_-TM and mutants. A representative plot from three independent experiments is shown. **d** Dimerization via the G interface slows down the nucleotide exchange of MFN2_IM_ and MFN1_IM_. The initial part of each fluorescence trace was magnified. **e** Effect of MFN2_IM_(T129I) and MFN1_IM_(I108T) on the nucleotide exchange efficiency in the dimeric state. The initial part of each fluorescence trace was magnified and fitted to exponential function as shown in a white curve. **f** Surroundings of MFN2-Thr129 and MFN1-Ile108 in the dimerization form. **g** Model for comparison between MFN2 and MFN1 in G domain dimerization. Note the tighter G interface of MFN2 and its sustained dimerization in the GDP-bound state. Pi denotes phosphate group. **h** Mitochondrial elongation assay with quantification for wild-type MFN2 and G interface mutants. Representative images are shown. *Mfn*1/2-null MEFs were transduced with retrovirus expressing 16× Myc-tagged WT Mfn2 or G interface mutants. Actin was used as a loading control. A lysate from WT MEFs is shown for comparison. For each construct, 100 cells were scored in biological triplicate. Error bars indicate s.e.m. Scale bars, 10 μm. Source data are provided as a Source Data file

The *trans* interactions were verified by single-point mutagenesis analysis. RALS analysis indicated that, except for MFN2_IM_(V273D) which completely abolished dimerization in the transition state, most of these mutants only led to partial or negligible destabilization of MFN2_IM_ dimer (Fig. 4a, Supplementary Fig. 6c). The resistance of the G interface to point mutations is in agreement with the stability of the MFN2_IM_ dimer. Analytical ultracentrifugation (AUC) assay revealed that the MFN2_IM_ dimer indeed has a much stronger *trans* association than MFN1_IM_ in the presence of ${\mathrm{GDP}} \bullet {\mathrm{BeF}}_{3}^{-}$, reflected by an increased dimer fraction at an equal protein concentration (Fig. 4b). We then performed a spectroscopic tethering assay. MFN2_IM_ or MFN1_IM_ were fused with MFN2 or MFN1 transmembrane domain (MFN2_IM_-TM or MFN1_IM_-TM), and reconstituted to liposomes (Supplementary Fig. 6d, e). The tethering of the proteoliposomes caused solution turbidity, which was monitored by measuring the light absorbance at a wavelength of 405 nm. Once GTP was added, MFN2_IM_-TM tethered liposomes in a significantly higher efficiency as compared with MFN1_IM_-TM (Fig. 4c).

To examine how G domain dimerization affects GTP turnover rate, we designed a nucleotide exchange assay by instantaneously mixing preincubated MFN1/2_IM_-GDP (monomeric) or MFN1/2_IM_-${\mathrm{GDP}} \bullet {\mathrm{BeF}}_{3}^{-}$ (dimeric) with fluorescently-labeled GTP (mant-GTP) using stopped-flow. The initial slope of the fluorescent increase indicates the efficiency of mant-GTP reload. Although MFN2_IM_ bound more mant-GTP over time due to a higher affinity to GTP as compared with MFN1_IM_, the mant-GTP reload to MFN2_IM_ was much slower. G domain dimerization greatly reduced the mant-GTP reload rate of both MFN2_IM_ and MFN1_IM_ (Fig. 4d). MFN2_IM_(T129I) and MFNI_IM_(I108T) showed promoted and reduced mant-GTP reload rates compared with wild-type proteins in ${\mathrm{GDP}} \bullet {\mathrm{BeF}}_{3}^{-}$ condition, respectively (Fig. 4e), suggesting that the Thr/Ile difference also plays an important role in G domain dimerization. In consistent with this, swapping MFN1-Ile108/MFN2-Thr129 elevated tethering activity for MFN1_IM_ and drastically reduced it for MFN2_IM_ (Fig. 4c). A structural explanation is that, compared with MFN1-Ile108, MFN2-Thr129 is more favored in the polar G interface for maintaining dimerization (Fig. 4f). Whether interrupting dimerization or not, the G interface mutants of MFN2_IM_ had comparable GTP turnover with wild type (Supplementary Fig. 6f). Altogether, the tight *trans* interaction within MFN2 dimer is likely to confine the conformational dynamics of switch I and prevent the reload of GTP, which ultimately accounts for the low GTP turnover rate (Fig. 4g). On the other hand, living cells seem quite sensitive to mutations in the G interface, as mutants I126D, N161A, and K307A were highly defective in mitochondrial elongation assays, although some residual activity remained (Fig. 4h).

### MFN2 interacts with MFN1 via the G interface

**Fig. 5:**
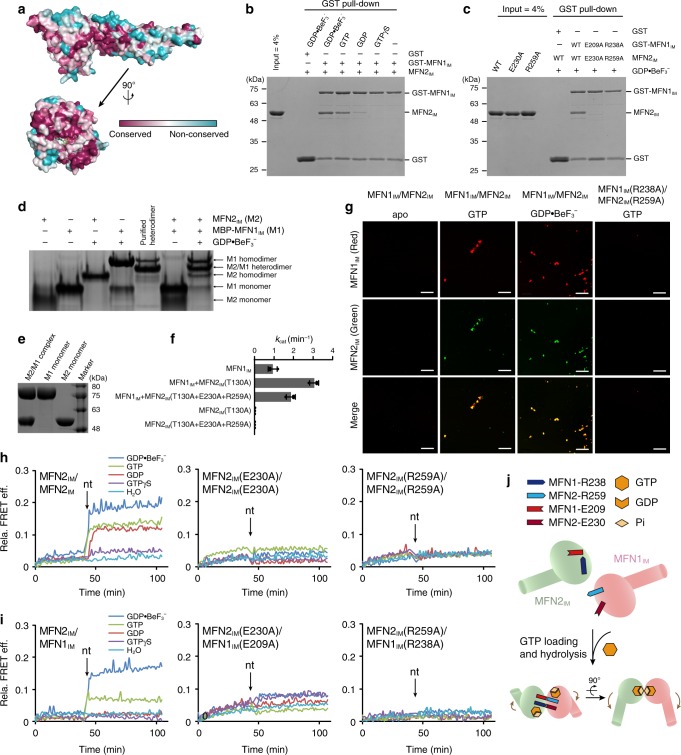
MFN2 interacts with MFN1 via G domain. **a** Surface conservation analysis reveals that the G interface is a highly conserved area in mitofusins. **b** Pull-down assays showing the interaction between MFN2_IM_ and GST-tagged MFN1_IM_ in different nucleotide-loading conditions. **c** Pull-down assays showing that the interaction between MFN2_IM_ and MFN1_IM_ is dependent on the G interface. **d** Native PAGE result showing the association between MFN2_IM_ and MBP-fused MFN1_IM_. **e** SDS-PAGE showing the purified MFN2_IM_/MBP-MFN1_IM_ hetero-complex. **f** MFN2_IM_ stimulates GTP turnover of MFN1_IM_. Overall, 0.25 μM MFN1_IM_ and 2.5 μM MFN1_IM_ MFN2_IM_ mutants were used. **g** Liposome tethering assay for wild-type MFN2_IM_/MFN1_IM_ and G interface mutants. Representative images from five independent experiments are shown. Scale bar, 50 μm. **h** FRET experiment showing the G domain dimerization-dependent conformational change of MFN2_IM_ in different nucleotide-loading conditions. The time point for the addition of corresponding nucleotides is indicated by an arrow for each panel. nt denotes nucleotide. **i** FRET experiment showing the conformational change of MFN2_IM_ and MFN1_IM_ upon association via the G domains in GTP- and ${\mathrm{GDP}} \bullet {\mathrm{BeF}}_{3}^{-}$ loading conditions. **j** A model for the *trans* association between MFN2_IM_ and MFN1_IM_. Source data are provided as a Source Data file

Surface conservation analysis using 12 mitofusin sequences from zebra fish to human revealed that the G interface is a highly conserved area in mitofusins (Fig. 5a). Given the fact that MFN1 and MFN2 share many key residues for G domain dimerization, we postulated that the G interface is involved in the formation of mitofusin hetero-complexes. To explore this idea, we performed pull-down assays in different nucleotide-loading conditions. MFN2_IM_ substantially co-eluted with glutathione S-transferase (GST)-tagged MFN1_IM_ in the presence of GTP or ${\mathrm{GDP}} \bullet {\mathrm{BeF}}_{3}^{-}$. GDP also led to weak association between the two proteins, but this hetero-association was not observed in apo or GTPγS-bound conditions (Fig. 5b). The G interface mutants MFN2_IM_(E230A) and MFN2_IM_(R259A) failed to interact with their MFN1_IM_ counterparts (Fig. 5c). When maltose-binding protein (MBP)-fused MFN1_IM_ and His_6_-tagged MFN2_IM_ were mixed together with ${\mathrm{GDP}} \bullet {\mathrm{BeF}}_{3}^{-}$, the MFN1_IM_–MFN2_IM_ heterodimer was efficiently formed and could be stably purified (Fig. 5d, e). The hydrolysis-deficient MFN2_IM_(T130A) was able to stimulate the GTP turnover of MFN1_IM_ in a similar manner to MFN1_IM_ self-stimulation^32^, and the introduction of two G interface mutations significantly weakened this effect (Fig. 5f). Liposomes that carry MFN1_IM_ fused with a transmembrane domain of Sac1 (MFN1_IM_-TM^Sac1^) were able to tether MFN2_IM_-TM^Sac1^-associated liposomes in the presence of GTP or ${\mathrm{GDP}} \bullet {\mathrm{BeF}}_{3}^{-}$, and the tethering was disrupted by the G interface mutants (Fig. 5g).

MFN1_IM_ was shown to have conformational dynamics coupled to the GTP turnover cycle^33^. In the transition state, the MFN1_IM_ dimer bends via the hinge 2, and the two HD1s move close to each other. This domain rearrangement can be monitored by FRET changes between labeled residues at appropriate positions. To understand whether MFN2_IM_ also has this feature, we introduced Cy3/Cy5 labels to MFN2_IM_ at the distal end of HD1 (Supplementary Fig. 7a–d), and traced the ensemble FRET in different nucleotide-loading states. Addition of GTP, ${\mathrm{GDP}} \bullet {\mathrm{BeF}}_{3}^{-}$, or GDP induced immediate FRET increase, whereas G interface mutants had no FRET change in all conditions (Fig. 5h). Similarly, mixture of differentially labeled MFN1_IM_ and MFN2_IM_ also exhibited FRET increase when GTP or ${\mathrm{GDP}} \bullet {\mathrm{BeF}}_{3}^{-}$ was supplied, and this FRET increase was apparently dependent on the G interface (Fig. 5i). These results confirmed that MFN2 confers domain rearrangement, and can associate with MFN1 via the G interface in a nucleotide-dependent manner (Fig. 5j).

### Structural and biochemical investigations of CMT2A mutations

**Fig. 6:**
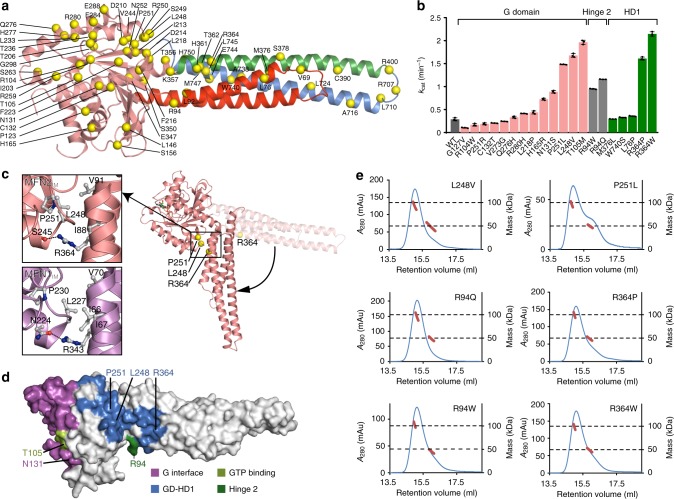
Characterization of CMT2A-related mutants. **a** Overview of the CMT2A-mutation sites on MFN2. The CMT2A-related single-point mutation sites are specified as yellow spheres on MFN2_IM_ structure colored as in Fig. 1b. **b** GTP turnover rates of selected CMT2A-related MFN2_IM_ mutants in comparison with wild-type MFN2_IM_. Locations of these mutants are color-specified. **c** CMT2A-mutation sites on the putative G domain-HD1 contact of MFN2 in the transition state. The structural model of rearranged MFN2_IM_ is generated base on the MFN1_IM_-${\mathrm{GDP}} \bullet {\mathrm{BeF}}_{3}^{-}$ structure (PDB code 5YEW). CMT2A-related residues and their potential interaction partners are presented as ball-and-stick models. **d** Functional zones of MFN2 and the involved CMT2A-mutation sites. **e** Dimerization property of CMT2A-mutants on the putative G domain-HD1 interface in the presence of ${\mathrm{GDP}} \bullet {\mathrm{BeF}}_{3}^{-}$. Source data are provided as a Source Data file

Mutations in the *Mfn2* gene are main cause of CMT2A. Most of these mutations result in single amino substitutions that are mainly distributed in the G domain and HD1. Although some CMT2A-related MFN2 mutations have been studied based on MFN1 structures^31,33^, more accurate conclusions require comprehensive analysis using a *bona fide* human MFN2 structure as the two mitofusins display substantial difference in the organization of G interface and GTPase activity. We plotted reported CMT2A mutations on the MFN2_IM_ structural model. The majority of them are on the surface of the protein (Fig. 6a). For example, the frequently mutated residues Arg104, Thr105, Gln276, and Arg280 are distributed around the G interface (Supplementary Fig. 8a). Arg94 is neighboring the hinge 2 linking G domain and HD1 (Supplementary Fig. 8b). Arg364 and Trp740 are on the surface of HD1 (Supplementary Fig. 8c). These surface-exposed disease-related residues are not involved in the intramolecular interactions, except that Arg104 forms a salt bridge with Asp204 in the GDP-bound state (Supplementary Fig. 8d). Overall, most of these mutations are not likely to severely disrupt folding of MFN2, as was corroborated by circular dichroism (CD) analysis (Supplementary Fig. 8e).

To explore the biochemical influence of these mutations, we tested 20 MFN2_IM_ constructs, each with a CMT2A-related mutation distributed in various regions of MFN2. Interestingly, over half of the mutants showed elevated GTPase activity compared with wild type, suggesting that slow GTP turnover is a crucial attribute for MFN2’s physiological function. Of these mutants, T105M and N131S are either on the P-loop or Switch I, and thus their direct influence on GTP turnover is intuitive. In contrast, L248V and P251L on the G domain, R94W and R94Q on the hinge 2, as well as R364P and R364W on HD1 are all distant from the catalytic site but strongly promoted GTP turnover (Fig. 6b). By referring to the ${\mathrm{GDP}} \bullet {\mathrm{BeF}}_{3}^{-}$-bound structure of MFN1_IM_^33^, we noted that Leu248, Pro251, and Arg364 are likely involved in the putative interface between the G domain and the reoriented HD1 (Fig. 6c, d). For example, Arg343 in MFN1, which is equivalent to MFN2-Arg364, forms a hydrogen bond with Asn224. Mutagenesis of these residues would disrupt this interface, thereby intervening the relative movement of G domain and HD1. R94W and R94Q are also expected to disturb the conformational dynamics of MFN2. These six mutants did not significantly perturb dimerization via the G interface (Fig. 6e), while some of their counterparts on MFN1 do^33^. This highlights a direct mechanistic coupling between the GTPase activity and domain rearrangement for MFN2 regardless of nucleotide-dependent dimerization, and may be relevant to a recent observation that a R364W-like mutation causes excessive mitochondrial fusion in a *Drosophila* model^39^.

**Fig. 7:**
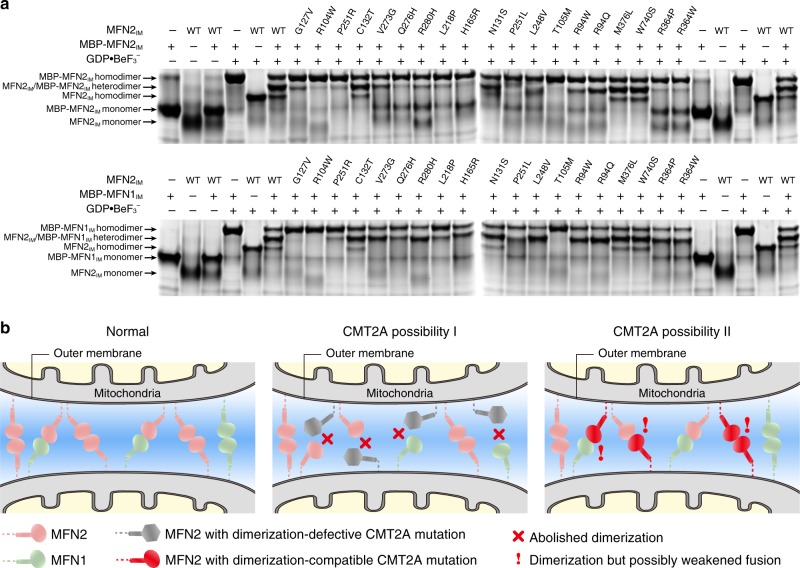
Interaction between CMT2A-related mutants and wild-type MFN1/2_IM_. **a** G domain-mediated association of CMT2A-related MFN2_IM_ mutants with MBP-fused MFN2_IM_ (upper) or MBP-MFN1_IM_ (lower) tested by native PAGE. **b** Schematic drawing showing the two causes of CMT2A onset that may be derived from different MFN2 mutations. Source data are provided as a Source Data file

While some CMT2A-related mutations occurring at the G interface (R104W, T105M, G127V) prevent the dimer formation in the transition state of GTP hydrolysis, other mutants could still dimerize (Supplementary Fig. 8f). To further test whether they are able to associate with wild-type mitofusins via G domains, we individually mixed the mutants with either MBP-fused MFN2_IM_ or MFN1_IM_ in the presence of GDP•BeF_3_^−^. Native PAGE analysis indicated that except those on the G interface, the majority of the CMT2A-related mutants efficiently heterodimerized with wild-type MFN2_IM_ or MFN1_IM_ (Fig. 7a). The R94W mutant, as an example, even formed no homodimers but only heterodimers with wild-type MFN2 or MFN1, which coincides with a previous co-immunoprecipitation study in human U2OS cells^40^. This implicates that corresponding mutations may have a dominant negative effect on the occurrence of CMT2A. The structural and biochemical information of available CMT2A-related mutations is summarized in Supplementary Table 1.

## Discussion

MFN2 and its homologs play pivotal roles in mitochondrial homeostasis and cell metabolism from yeast to human. Our study reveals the structural and biochemical features of this key molecule that underlie its divergent functions. MFN2 possesses most of the traits of the dynamin superfamily such as domain organization and G domain-mediated dimerization. However, unlike almost all other members (including human MFN1) with dimerization-stimulated GTPase activity that entails frequent attaching–detaching cycles of the G domains coupled with multiple rounds of GTP hydrolysis, MFN2 has a hydrolyze-but-not-dissociate feature. Keeping basal (low) GTP turnover seems crucial for the cellular function of MFN2, as many GTPase activity-promoting mutants are associated with CMT2A onset. Of these mutants, T105M and P251L have been previously shown to lack fusogenic activity^41^. The more stable G domain dimerization and potent tethering activity, as compared with human MFN1 in our experiment, imply that MFN2 may act as a GTP-regulated molecular sticker for mitochondria. GTP-loaded MFN2 molecules may be able to establish enduring trans anchoring of juxtaposing mitochondria throughout hydrolysis cycle. Although MFN2 has been previously reported to bear weaker tethering activity than MFN1^11^, the isolated MFN2 used in the tethering experiment may already have been loaded with host GDP. Thus, it is tempting to speculate that MFN2 molecules serve as initial mitochondrial anchors in the beginning of the fusion process. The free and vigorous relative movement between HD1 and HD2 via hinge 1, as found also in dynamin-like MxA and bacterial DLPs^42–44^, may draw the tethered mitochondrial segments together. This proposed mechanism is economic in GTP consumption, thereby ensuring that mitochondrial tethering can happen at low local MFN2 or GTP concentrations. Given the tight G interface of MFN2, it is possible that external partners, such as the Smad2-RIN1 complex that are reported to promote GTP turnover of MFN2^45^, help with G domain dissociation after GTP hydrolysis. Other cellular factors may also participate in the regulation of MFN2: the potential of binding Ca^2+^ and citrate found in the crystal structure implies a possibility of physiological relevance between MFN2 and these molecules. It has been recently reported that Ca^2+^ affects mitochondrial fusion by inhibiting MFN1 assembly^46^, and citrate is known to be transported to cytoplasm from Krebs cycle and modulate acetyl-CoA carboxylase for fatty acid metabolism^47^. Can they somehow influence OMM fusion through direct interaction with MFN2 in living cells? It may be worthwhile to explore these ideas.

A following question is whether the tethering efficiency of MFN2 translates to high fusogenic activity. On one hand, tight dimerization and resulting low GTP turnover rate of MFN2 may not favor mitochondrial fusion, as the fusion event requires continuous GTP hydrolysis cycles. Firmly *trans*-associated MFN2 molecules stacking between two segments of mitochondrial outer membrane may even prevent their contact. On the other hand, if these *trans*-associated MFN2 molecules are organized in so-called docking ring complexes as reported earlier in an in vitro assay for yeast Fzo1^48^, mitochondrial fusion may be promoted. We used internally truncated MFN1/2 variants in liposome tethering assays, and they lack the HD2 region which is indispensible for fusion. Although previous studies showed that MFN1/2 (or Fzo1) are able to fuse mitochondria in vitro by using mitochondria isolated from mouse embryonic fibroblasts (MEFs)^49^ or yeast^48^, one cannot exclude the possibility that these mitochondria carry other adapters (specific lipids or proteins) that are essential for fusion. To understand whether MFN2 (or MFN1) on its own can efficiently catalyze mitochondrial outer membrane fusion, an in vitro fusion assay using full-length mitofusin and clean mitochondria (or liposomes) will be needed.

The difference between human MFN1 and MFN2 in GTP turnover efficiency largely derives from the Ile108/Thr129 variance, which can lead to difference in the flexibility of switch I and also in the stability of G interface. By careful sequence screening, we found that this single amino acid variance of MFN1/2 only exists in primates (Supplementary Fig. 4b). In this context, the particularly high GTPase activity of human MFN1, as compared with other mitofusins, suggests that primates may have special needs for mitochondrial fusion. In line with these ideas, MFN1 in nonprimate mammals is likely to share more functional redundancy with MFN2 as they do not display the Ile/Thr variance in switch I. As currently there is no MFN1 knockout data for primates, the question why MFN1 mutations are not found to be clearly related with human diseases, say CMT2A, may have two distinct answers: (i) primate MFN2 is unable to compensate for defective MFN1, and detrimental mutations in MFN1 cause death in early embryonic development; (ii) primate MFN2 can efficiently substitute defective MFN1 but not vice versa, therefore only defects in MFN2 lead to disease. Given this functional complexity, the physiological role of MFN1 in humans needs to be dissected in more detail, and proper animal models are required.

The heterodimerization between human MFN2 and MFN1 found in our study is likely to occur in *trans* and compete with homotypic MFN1/MFN2 associations during mitochondrial fusion as the two types of interactions are both mediated by the G interface. According to the native PAGE results, the tendency of MFN1–MFN2 heterodimerization in vitro is even stronger than that of homodimerization (Figs. 5d, 7a). If this is true also for mouse mitofusins, it may partly explain the previous observation that heterotypic MFN1/MFN2 *trans* complexes show greater fusogenic efficacy in MEFs as compared with homotypic MFN1 or MFN2 complexes^49^. It is important to investigate the particular physiological role of this heterodimerization in OMM fusion and ER–mitochondria tethering in vivo in the future, and our results contribute to the development of more efficacious methodologies for this purpose.

Based on our CD spectroscopy experiments, the majority of reported CMT2A-related missense mutations do not seem highly detrimental to protein folding. Some of these mutants may even preserve a certain level of functionality in OMM fusion. These mutations can be mapped to at least four functional zones of the molecule, including (I) the nucleotide-binding site (the P-loop, Switch I/II and G4 motif), (II) G interface, (III) hinge 2, and (IV) G domain-HD1 interface (Fig. 6d, Supplementary Table 2). The consequence of these mutations in these zones can therefore be predicted from the biochemical feature of MFN2. For example, R104W of the nucleotide-binding site, R94W of hinge 2 and R364P of the G domain-HD1 interface all cause severe CMT2A phenotype, whereas the dominant L745P and Q276R mutations, which are beyond the above four functional zones, only lead to mild or moderate disease conditions^50^. It is also possible that, mutations of residues beyond these zones but involved in critical interaction with other partners of MFN2, also results in severe CMT2A phenotype.

Different mechanisms for MFN2-related CMT2A occurrence are also expected on the intermolecular level. The dimerization-deficient T105M mutant is highly defective in promoting mitochondrial fusion^41^, but it is less likely to affect the functionality of wild-type MFN1 and MFN2, at least in terms of G domain association. Consequently, the development of the CMT2A phenotype in T105M patients are likely due to reduced cellular level of normal MFN2. This mechanism possibly also applies to *MFN2* mutations that lead to unfolded or truncated proteins. On the other hand, those mutants that are still able to dimerize and retain residual fusogenic activity, as compared with MFN2(T105M), tend to have a dominant negative effect through hijacking normal MFN1 and allelic MFN2, which eventually results in a pervasively weakened MFN2 and MFN1 activity (Fig. 7b). In this case, the level of retained fusogenic activity of the mutant is a key determinant for the onset of the disease. Altogether, this information will be valuable for the diagnosis and prognosis of CMT2A cases, and for the development of personalized treatments targeting particular MFN2 mutations.

## Methods

### Protein expression and purification

The engineered MFN2 construct (MFN2_IM_) was cloned into a modified pET-28 vector. Details of this construct are illustrated in Fig. 1a. All mutants were generated by site-directed mutagenesis. Primer sequences for amplification and cloning here are provided in Supplementary Table 2 (primers 1–4). Recombinant MFN2_IM_ proteins containing an N-terminal His_6_-tag followed by a cleavage site for PreScission protease (PSP) were expressed in *E. coli* *Rosetta* (DE3) cells (Invitrogen). Transformed bacteria were cultured at 37 °C before induced with 0.1 mM isopropyl-1-thio-β-d-galactopyranoside (IPTG) at an OD_600 nm_ of 0.6, and grown for 20–24 h at 17–18 °C in Terrific Broth medium. Initially, human MFN2_IM_ was purified following our previous protocol for MFN1_IM_^32^, which was used for obtaining GDP-bound MFN2_IM_ crystals. To remove host-derived nucleotides bound to MFN2_IM_, cells were lysed in 50 mM HEPES, pH 7.5, 1 M NaCl, 30 mM imidazole, 2.5 mM β-mercaptoethanol (β-ME), 1 μM DNase I, and 1 mM phenylmethanesulfonylfluoride (PMSF) using a cell disruptor (JNBIO) and subjected to centrifugation at 40,000 *g* for 50 min. The supernatant was filtered and applied to an Ni-NTA (first Ni-NTA) column (GE Healthcare) equilibrated with buffer A containing 20 mM HEPES, pH 7.5, 1 M NaCl, 30 mM imidazole, and 2.5 mM β-ME. After washed with buffer A, protein was eluted with 20 mM HEPES, pH 7.5, 1 M NaCl, 300 mM imidazole, and 2.5 mM β-ME. Eluted proteins were incubated with 20 μg glutathione S-transferase (GST)-fused PSP to remove the His_6_-tag and dialyzed overnight against buffer B containing 20 mM HEPES, pH 7.5, 800 mM NaCl, 2.5 mM β-ME. After dialysis, PSP was removed by a GST column. The protein was reapplied to a second Ni-NTA column equilibrated with buffer B and eluted with buffer A, and further purified by gel filtration chromatography (Superdex 200 16/60 column, GE Healthcare) in buffer C containing 20 mM HEPES, pH 7.5, 150 mM KCl, 5 mM MgCl_2_, and 1 mM dithiothreitol (DTT). For nucleotide-free MFN2_IM_ used in crystallization experiments, KCl was substituted by NaCl in buffer C during gel filtration.

His_6_-tagged MFN1_IM_ and mutants were purified as previously described^32^. Cells expressing MFN1_IM_ or mutants were lysed in 50 mM HEPES, pH 7.5, 400 mM NaCl, 5 mM MgCl_2_, 30 mM imidazole, 1 μM DNase I, 1 mM PMSF, and 2.5 mM β-ME and subjected to centrifugation at 40,000 *g* for 1 h. The supernatant was filtered and applied to a Ni-NTA (first Ni-NTA) column (GE Healthcare) equilibrated with binding buffer D containing 20 mM HEPES, pH 7.5, 400 mM NaCl, 5 mM MgCl_2_, 30 mM imidazole, and 2.5 mM β-ME. After washed with binding buffer D, proteins were eluted with elution buffer containing 20 mM HEPES, pH 7.5, 400 mM NaCl, 5 mM MgCl_2_, 300 mM imidazole, and 2.5 mM β-ME. Eluted proteins were incubated with 20 μg GST-fused PSP to remove the His_6_-tag and dialyzed overnight against binding buffer E containing 20 mM HEPES, pH 7.5, 400 mM NaCl, 5 mM MgCl_2_, and 2.5 mM β-ME. After dialysis, PSP was removed using a GST column. The protein was reapplied to a second Ni-NTA column equilibrated with binding buffer E. Binding buffer D was used to elute the proteins which were subsequently loaded onto a Superdex 200 16/60 column (GE Healthcare) equilibrated with gel filtration buffer C. Cell lysis and protein purification were both performed at 4 °C.

*E. coli* cells expressing GST- and MBP-fused MFN1_IM_ (recombinant in pGEX-6p-1 vector or a modified MBP vector, a gift from Professor Tengchuan Jin), as well as corresponding mutants, were cultured and lysed in similar protocols as His_6_-tagged MFN1_IM_ construct, but the lysis buffer contains no imidazole. In the affinity chromatography experiments, elution buffer contains either 15 mM reduced glutathione (GSH) for GST-fused MFN1_IM_, or 10 mM maltose for MBP-fused MFN1_IM_. The protein was purified by gel filtration chromatography in buffer C. All cell lysis and protein purification performances were carried out at 4 °C. Primer sequences for amplification and cloning here are provided in Supplementary Table 2 (primers 27 and 32 for pGEX-6p-1 vector; primers 5–8 for MBP vector).

For transmembrane domain (TM)-fused MFN1_IM_ or MFN2_IM_ used in spectroscopic tethering assay. The original residues 365–694 of MFN1 were replaced with the TM of codon-optimized MFN1 (residues 596–628) flanked by amino acid sequences SGSGSGGS (N-terminal) and GSGS (C-terminal) using overlap PCR. The original residues 401–705 of MFN2 were replaced with the TM of MFN2 (residues 615–647) flanked by SAASA (N-terminal) and ASAA (C-terminal). The fragments were individually cloned into the pGEX-6p-3 vector. Primer sequences for amplification and cloning here are provided in Supplementary Table 2 (primers 9–20). All point mutations were generated by site-directed mutagenesis. MFN1_IM_-TM, MFN2_IM_-TM and corresponding mutants were expressed and purified as previously described^33^. Plasmids expressing MFN1_IM_-TM or MFN2_IM_-TM were transformed into *E. coli BL21* (DE3). Cells were grown to an OD_600 nm_ of 0.8, induced with 0.3 mM IPTG for 24 h at 16 °C, and harvested by centrifugation. The pellets were resuspended in buffer F containing 25 mM HEPES, pH 7.4, 150 mM NaCl, 10% glycerol, 1 mM EDTA, and 2 mM β-ME. Membranes were then pelleted by centrifugation at 110,000 *g* for 1 h, and dissolved by 1% Fos-choline-12 (Anatrace) in buffer F, and insoluble components were cleared by centrifugation. The recombinant protein was isolated by glutathione Sepharose (GE Healthcare), washed twice with buffer F containing 0.1% Triton X-100 (Anatrace), and the GST-tag was cleaved before reconstitution.

For constructs used in visual tethering assay, the TM of Sac1 (residues 523–580) was inserted between α3^H^ and α4^H^ of MFN1_IM_ (MFN1_IM_-TM^Sac1^) or MFN2_IM_ (MFN2_IM_-TM^Sac1^) by overlapping PCR, and the fragments were individually cloned into pGEX-6p-1 vector. Primer sequences for amplification and cloning here are provided in Supplementary Table 2 (primers 21–32). MFN1_IM_-TM^Sac1^, MFN2_IM_-TM^Sac1^ and corresponding mutants were expressed and purified as previously described^51^. Proteins were expressed in *E. coli Rosetta* (DE3) cells for 16 h at 17–18 °C. Collected cells expressing MFN1/2_IM_-TM^Sac1^ or its mutants were lysed in 50 mM HEPES, pH 7.5, 150 mM NaCl, 30 mM imidazole, 10% glycerol, 2% Triton X-100, 1 µM DNase I, 1 mM PMSF, and 2.5 mM β-ME and centrifugated at 40,000 *g* for 30 min. After incubation at 4 °C for 1 h, the supernatant was subjected to further centrifugation at 40,000 *g* before filtered and applied to a GST column (GE healthcare) which is equilibrated with binding buffer G containing 50 mM HEPES, pH 7.5, 150 mM NaCl, 30 mM imidazole, 10% glycerol, 0.1% Triton X-100, and 2.5 mM β-ME. Target proteins were eluted with buffer G supplied with 15 mM GSH. Target proteins with GST-tags removed were reapplied to a GST column after dialysis using buffer G. Eluted target proteins were loaded onto a Superdex 200 16/60 column equilibrated with 20 mM HEPES, pH 7.5, 150 mM NaCl, 5% glycerol, 0.1% Triton X-100 and 1 mM DTT and collected. For MFN2_IM_-TM^Sac1^ or its mutants, 1 M NaCl instead of 150 mM NaCl was used in buffer G.

### Protein crystallization

MFN2_IM_ constructs were crystallized via hanging drop vapor diffusion by mixing equal volumes of protein (~20 mg ml^−1^) and reservoir solution. Crystals of nucleotide-free MFN2_IM_ grew from 0.19 M calcium chloride, 95 mM HEPES, pH 7.5, 26.6% PEG400, 5% glycerol at 4 °C. MFN2_IM_(T111D) was crystallized in 0.16 M calcium acetate, 0.08 M sodium cacodylate pH 6.5, 14.4% PEG 8000, 20% glycerol at 18 °C. Crystals of GDP-bound MFN2_IM_ were grown from 0.2 M lithium citrate tribasic tetrahydrate, and 35% glycerol ethoxylate at 18 °C using MFN2_IM_ purified with previous protocol for MFN1_IM_^32^.

### Structure determination

X-ray diffraction datasets were collected at beamlines BL17U1 and BL19U1 of Shanghai Radiation Facility (SSRF)^52^ and processed with the XDS suite^53^. Structures of MFN2_IM_ were solved by molecular replacement using MOLREP^54^ with truncated MFN1_IM_ as the search model. Models were built with COOT^55^ and refined with Refmac^56^ and Phenix^57^. Structural validation was carried out using MolProbity^58^. Structural illustrations were prepared using the PyMOL Molecular Graphic Systems (version 0.99, Schrödinger LLC; http://www.pymol.org/). The Ramachandran statistics determined by PROCHECK^59^ are as follows: 97.7% in favored region, 2.3% allowed, 0 outlier for apo MFN2_IM_; 98.3% favored, 1.5% allowed, 0.2% outlier for MFN2_IM_(T111D) and 98.6% favored, 1.4% allowed, 0 outlier for GDP-bound MFN2_IM_.

### Nucleotide-binding assay

The equilibrium dissociation constants for nucleotide-free MFN2_IM_, MFN1_IM_ and indicated mutants to guanine nucleotides were determined by ITC at 25 °C using a MicroCal ITC200 (Malvern) in the buffer containing 20 mM HEPES, pH 7.5, 150 mM KCl, 5 mM MgCl_2_. A total of 1–2 mM nucleotides were used to titrate 80 μM protein. Resulting heat changes upon each injection was integrated and the values were fitted to a standard single-site binding model using Origin7.

### GTP hydrolysis assay

GTP hydrolysis assays were performed at 37 °C as previously described^32^ in buffer containing 20 mM HEPES, pH 7.5, 150 mM KCl, 5 mM MgCl_2_, and 1 mM DTT. Reactions were initiated by the addition of protein to the final reaction solution. At different time points, reaction aliquots were 20-fold diluted in buffer C and quickly transferred into liquid nitrogen. Nucleotides in the samples were separated via a reversed-phase Hypersil ODS-2 C18 column (250 × 4.6 mm, Thermo), with 10 mM tetrabutylammonium bromide, 100 mM potassium phosphate, pH 6.5, and 7.5% acetonitrile as running buffer, where denatured proteins were blocked by a C18 guard column (Thermo). Nucleotides were detected by absorption at 254 nm and quantified by integration of the corresponding peaks. The GTP hydrolysis rates derived from a linear fit to the initial phase of the reaction (<40% GTP hydrolyzed) were plotted against the protein concentrations. For measuring stimulated GTP turnover of MFN2_IM_ and MFN1_IM_, proteins at concentrations of 0.5, 1, 2, 4, 8, 16, and 32 μM were individually mixed with 1–2 mM GTP. For measuring stimulated GTP turnover of MFN1_IM_ by hydrolysis-deficient MFN1_IM_ or MFN2_IM_ constructs, 0.25 μM MFN1_IM_ and 1 mM GTP were mixed with 2.5 μM MFN2_IM_(T130A) or MFN2_IM_(T130A/E230A/R259A). For other experiments, 32 μM protein and 2 mM GTP were used.

### Fast kinetics assay

To determine the GDP off-rate, 10 μM protein was preincubated with 2 μM N-methyl-3′-O-anthranoyl (mant-)GDP (Jena Bioscience) for 2 h before rapidly mixed with 1 mM unlabeled GDP in a buffer containing 20 mM HEPES, pH 7.5, 150 mM KCl, 5 mM MgCl_2_ and 1 mM DTT, and the fluorescence signal (excitation wavelength 355 ± 4 nm) was instantaneously recorded using a Chirascan spectrometer equipped with an SX20 Stopped-Flow accessory (Applied Photophysics). Three injections were individually and consecutively carried out, and the readouts were averaged to yield a time-dependent fluorescence change diagram. The GDP off-rates were derived from fitting the fluorescence traces with single exponential function.

For mant-GTP exchange assay, 5 μM proteins were preincubated with 50 μM GDP or 50 μM GDP•BeF_3_¯ (50 μM GDP, 50 μM BeSO_4_, and 500 μM NaF) at room temperature for 2 h before rapidly mixed with 2 μM mant-GTP in a buffer containing 20 mM HEPES, pH 7.5, 150 mM KCl, 5 mM MgCl_2_, and 1 mM DTT.

### RALS

A coupled RALS-refractive index detector (Malvern) was connected in line to an analytical gel filtration column Superdex 200 10/300 to determine absolute molecular masses of the applied protein samples. 50 μM purified protein was incubated with or without 500 μM corresponding ligand for 2 h at room temperature before applied to the column equilibrated with 20 mM HEPES, pH 7.5, 150 mM KCl, 5 mM MgCl_2_, and 1 mM DTT. Data were analyzed with the OMNISEC software. All experiments were repeated at least twice and the data showed satisfying consistency.

### Mitochondrial elongation assay

To examine the effect of point mutations, MFN2–Myc variants were expressed in *Mfn1*/*2*-null MEFs^10^ from the pQCXIP retroviral vector. The cell line is free of mycoplasma and has been authenticated by genotyping with PCR to confirm deletion of the *Mfn1* and *Mfn2* genes. Point mutants in mouse *Mfn2* were constructed by overlapping PCR with primers encoding the point mutation. For I126D, the 5′ region of the *Mfn2* cDNA was amplified with primers 33 and 34, and subcloned as a NotI/MfeI fragment. For N161A, two overlapping *Mfn2* fragments were amplified with primers 35–38 (N161A). The two fragments were combined, reamplified with primers 35 and 38, and subcloned as an MfeI/PsiI fragment. The same strategy was used to subclone K307A, except that primers 39 and 40 were used instead of primers 36 and 37. All primers used here are provided in Supplementary Table 2.

All mutations were confirmed by DNA sequencing. The types and positions of the residues mutated in this study are all consistent between human and mouse mitofusins. Retroviral supernatants were produced from 293T cells transfected with the retroviral vector and the packaging plasmid pCLEco. *Mfn*1/2-null MEFs stably expressing mito-DsRed were maintained in Dulbecco’s Modified Eagle’s Medium supplemented with 10% fetal bovine serum and penicillin/streptomycin at 37 °C and 5% CO_2_. After retroviral transduction of *Mfn*1/2-null MEFs, puromycin selection was applied for 2 days. Cells were plated onto eight-well chambered slides for analysis. Western blot analysis with the 9E10 antibody against Myc (DSHB, AB_2266850) was performed to confirm proper expression of the MFN2 variant. Mitochondrial morphology was scored by analysis of mito-DsRed as described previously^10^. Cells were imaged with a Plan NeoFluar ×63 objective on a Zeiss 410 laser scanning confocal microscope (Carl Zeiss MicroImaging, Inc.).

### Analytical ultracentrifugation

Twenty micromoles of MFN1_IM_ and MFN2_IM_ mixed with 1 mM GDP, 1 mM BeSO_4_, and 10 mM NaF were applied to AUC in a buffer containing 20 mM Tris, pH 8.0, 150 mM KCl, and 5 mM MgCl_2_. Sedimentation velocity experiments were performed with an An-60 Ti rotor at a speed of 145,000 *g* in a ProteomeLab XL-I Protein Characterization System (Beckman Coulter) at 20 °C. All interference data were processed according to a c(s) distribution model.

### Spectroscopic tethering assay

Purified MFN1_IM_-TM, MFN2_IM_-TM, and corresponding mutants were reconstituted into preformed liposomes (83.5:15:1.5 mol % POPC/DOPS/Rhodamine-DPPE) as previously described^33^. Two micromoles of protein was reconstituted into 2 mM liposome in buffer H (25 mM HEPES, pH 7.4, 150 mM KCl, 10% glycerol), and detergent was removed by Biobeads. Five millimoles of MgCl_2_ was added immediately before measuring the absorbance at 405 nm. Data were collected every 30 s using a Microplate Reader (Tecan). The absorbance before nucleotide addition was set to zero. To verify reconstitution efficiency, 30 µl proteoliposomes were mixed with 100 µl of 1.9 M sucrose and overlaid with 100 µl of 1.25 M sucrose and 20 µl buffer H supplied with 1 mM EDTA and 2 mM β-ME. After centrifugation in a Beckman TLS-55 rotor at 174,000 *g* for 75 min at 4 °C, 50 µl fractions were collected from the top of the gradient. Top and bottom fractions were analyzed by SDS-PAGE.

### Visual tethering assay

POPC, DOPS, Texas Red DHPE, or Oregon green 488 DHPE (ThermoFisher) were mixed in a molar ratio of 84.5:15:0.5 as previously described^51^. Overall, 0.3 μM MFN1_IM_-Sac1^TM^ or MFN2_IM_-Sac1^TM^ or corresponding mutants were reconstituted into 0.6 mM prepared liposomes with Texas Red DHPE or Oregon green 488 DHPE in a buffer containing 20 mM HEPES, pH 7.5, 150 mM NaCl, 5 mM KCl, 5% glycerol, and 2% β-ME. Proteoliposomes labeled with red and green dyes were 1:1 mixed, 1 mM ligands and 5 mM MgCl_2_ were subsequently added. Samples were incubated at 37 °C for 0.5 h before imaged by a confocal laser scanning microscopy (OLYMPUS FV1000).

### Surface conservation plot

Protein sequences of 24 mitofusins were aligned using Clustal Omega^60^ (https://www.ebi.ac.uk/Tools/msa/clustalo/). These sequences from 12 vertebrate species included MFN1 and MFN2 from *Homo sapiens* (UniProt accession Q8IWA4 and O95140, respectively), *Sus scrofa* (M3VH65 and F1RF76), *Bos taurus* (F1MPL1 and D7GLD0), *Equus caballus* (F6VTU8 and F6YL70), *Ovis aries* (W5QHQ0 and W5Q8M0), *Canis lupus familiaris* (F1Q1G1 and E2R3M9), *Oryctolagus cuniculus* (G1SEC8 and G1SU75), *Felis catus* (A0A1D5PYU0 and M3VU19), *Mus musculus* (Q811U4 and Q80U63), *Gallus gallus* (A0A1D5PER5 and E1BSH7), *Xenopus tropicalis* (Q28FR9 and F6QKB9), and *Danio rerio* (Q6PFP9 and A8WIN6), The alignment result and structure of MFN2_IM_-GDP complex were uploaded to the ConSurf online server^61^ (http://conseq.tau.ac.il) to compute conservation scores for the residues. Surface plot of MFN2_IM_ with conservation score-based coloring was generated using PyMOL Molecular Graphic Systems (version 0.99, Schröinger LLC; http://www.pymol.org/).

### Pull-down assay

Twenty micrograms of GST-tagged MFN1_IM_ was incubated with 60 μg MFN2_IM_ with or without 2 mM corresponding ligand for 2 h at 4 °C in a buffer containing 20 mM HEPES, pH 7.5, 150 mM NaCl, 5 mM MgCl_2_, and 1 mM DTT. The mixtures were sampled to 100 μg GST beads (GE Healthcare) and then washed with the same buffer. Proteins retained by the GST beads were eluted by extra 20 mM GSH before analyzed by SDS-PAGE.

### Native PAGE

Blue native PAGE technique was used to check the homo- and hetero-dimerization of MFN2_IM_. Ten micromoles of MBP-tagged MFN1_IM_/MFN2_IM_ was mixed with 10 μΜ untagged MFN1_IM_/MFN2_IM_ in the absence or presence of tenfold concentration of indicated nucleotides for 2 h at room temperature. The samples were then mixed with 5× loading buffer (1%[w/v] bromophenol blue, 50%[v/v] glycerol) and subjected to native PAGE analysis at 4 °C. Electrophoresis was performed at 100 V for 20 min in 4% stacking gel, and then 160 V for 70 min in separating gel. The gel was stained with Coomasie brilliant blue.

### FRET assay

MFN1_IM_(C156S + A696C), MFN2_IM_(C390S + A706C), and related mutants were labeled with fivefold concentration of fluorescent dye Cy3 or Cy5 (GE Healthcare) for 60 min at 4 °C in a buffer containing (20 mM HEPES, pH 7.5, 150 mM KCl, 5 mM MgCl_2_, and 0.5 mM Tris(2-carboxyethyl)phosphine (TCEP). Free dye was removed by gel filtration chromatography using a Superdex 200 10/30 column. Cy3 was excited at 537 nm, with peak emission at 570 nm. Cy5 fluorescence emission was detected at 667 nm. Cy3- and Cy5-labeled proteins (1 μΜ each) were mixed in a buffer containing 20 mM HEPES, pH 7.5, 150 mM KCl, 5 mM MgCl_2_, and 2% β-ME as previously described^33^. Fluorescence was measured once every 1 min in a flat black 96-well plate for 40 min, then 2 mM indicated nucleotide was added and the measurement continued for another 60 min. FRET traces were calculated as: FRET = *I*_Cy5_/(*I*_Cy3_ + *I*_Cy5_), where *I*_Cy3_ and *I*_Cy5_ are the instantaneous Cy3 and Cy5 fluorescence intensities, respectively.

### Circular dichroism

Proteins were diluted to 0.1 mg ml^−1^ in ddH_2_O and applied to CD measurements using a Chirascan spectrometer (Applied Photophysics) in quartz cuvettes with path length of 0.5 mm. Spectra were recorded from 180 to 260 nm at a bandwidth of 1 nm. All data were collected using a stop resolution of 1 nm and time per point of 0.5 s. A control spectrum obtained from the diluted buffer was subtracted from the original data. CD readouts were converted to mean residue ellipticity (M.R.E).

### Reporting summary

Further information on research design is available in the Nature Research Reporting Summary linked to this article.

## Supplementary information

Supplementary Information

Reporting Summary

Source Data

## Data Availability

Data supporting the findings of this paper are available from the corresponding author upon reasonable request. A reporting summary for this article is available as a Supplementary Information file. The source data underlying Figs. 2a, d, 4h, 5b–f, 6b, a and Supplementary Figs. 3e, 4a, 5f, 6e, f, 7b, c are provided as a Source Data file. The X-ray crystallographic coordinates and structure factor files for MFN2_IM_ structures have been deposited in the Protein Data Bank (PDB) under the following accession numbers: 6JFL for nucleotide-free MFN2_IM_, 6JFK for GDP-bound MFN2_IM_, and 6JFM for MFN2_IM_(T111D).

## References

[1] Shaw JM, Nunnari J (2002). Mitochondrial dynamics and division in budding yeast. Trends Cell Biol..

[2] Youle RJ, van der Bliek AM (2012). Mitochondrial fission, fusion, and stress. Science.

[3] Mishra P, Chan DC (2016). Metabolic regulation of mitochondrial dynamics. J. Cell Biol..

[4] Yaffe MP (1999). The machinery of mitochondrial inheritance and behavior. Science.

[5] Chan DC (2012). Fusion and fission: interlinked processes critical for mitochondrial health. Annu. Rev. Genet..

[6] Friedman JR, Nunnari J (2014). Mitochondrial form and function. Nature.

[7] Santel A, Fuller MT (2001). Control of mitochondrial morphology by a human mitofusin. J. Cell. Sci..

[8] Praefcke GJ, McMahon HT (2004). The dynamin superfamily: universal membrane tubulation and fission molecules?. Nat. Rev. Mol. Cell Biol..

[9] Chen H (2003). Mitofusins Mfn1 and Mfn2 coordinately regulate mitochondrial fusion and are essential for embryonic development. J. Cell Biol..

[10] Koshiba T (2004). Structural basis of mitochondrial tethering by mitofusin complexes. Science.

[11] Ishihara N, Eura Y, Mihara K (2004). Mitofusin 1 and 2 play distinct roles in mitochondrial fusion reactions via GTPase activity. J. Cell. Sci..

[12] Filadi R (2015). Mitofusin 2 ablation increases endoplasmic reticulum-mitochondria coupling. Proc. Natl Acad. Sci. US.

[13] de Brito OM, Scorrano L (2008). Mitofusin 2 tethers endoplasmic reticulum to mitochondria. Nature.

[14] Narendra D, Tanaka A, Suen DF, Youle RJ (2008). Parkin is recruited selectively to impaired mitochondria and promotes their autophagy. J. Cell Biol..

[15] Ziviani E, Tao RN, Whitworth AJ (2010). Drosophila parkin requires PINK1 for mitochondrial translocation and ubiquitinates mitofusin. Proc. Natl Acad. Sci. USA..

[16] Chen Y, Dorn GW (2013). PINK1-phosphorylated mitofusin 2 is a parkin receptor for culling damaged mitochondria. Science.

[17] Ngoh GA, Papanicolaou KN, Walsh K (2012). Loss of mitofusin 2 promotes endoplasmic reticulum stress. J. Biol. Chem..

[18] Sebastian D (2012). Mitofusin 2 (Mfn2) links mitochondrial and endoplasmic reticulum function with insulin signaling and is essential for normal glucose homeostasis. Proc. Natl Acad. Sci. USA..

[19] Misko A, Jiang S, Wegorzewska I, Milbrandt J, Baloh RH (2010). Mitofusin 2 is necessary for transport of axonal mitochondria and interacts with the Miro/Milton complex. J. Neurosci..

[20] Chen KH (2014). Role of mitofusin 2 (Mfn2) in controlling cellular proliferation. FASEB J..

[21] Schrepfer E, Scorrano L (2016). Mitofusins, from Mitochondria to Metabolism. Mol. Cell.

[22] Züchner S (2004). Mutations in the mitochondrial GTPase mitofusin 2 cause Charcot-Marie-Tooth neuropathy type 2A. Nat. Genet..

[23] Wang X (2009). Impaired balance of mitochondrial fission and fusion in Alzheimer’s disease. J. Neurosci..

[24] Lee S (2012). Mitofusin 2 is necessary for striatal axonal projections of midbrain dopamine neurons. Hum. Mol. Genet..

[25] Celardo I (2016). Mitofusin-mediated ER stress triggers neurodegeneration in pink1/parkin models of Parkinson’s disease. Cell Death Dis..

[26] Chen Y, Liu Y, Dorn GW (2011). Mitochondrial fusion is essential for organelle function and cardiac homeostasis. Circ. Res..

[27] Papanicolaou KN (2012). Mitofusins 1 and 2 are essential for postnatal metabolic remodeling in heart. Circ. Res..

[28] Bach D (2005). Expression of Mfn2, the Charcot-Marie-Tooth neuropathy type 2A gene, in human skeletal muscle: effects of type 2 diabetes, obesity, weight loss, and the regulatory role of tumor necrosis factor alpha and interleukin-6. Diabetes.

[29] Schneeberger M (2013). Mitofusin 2 in POMC neurons connects ER stress with leptin resistance and energy imbalance. Cell.

[30] Filadi R, Pendin D, Pizzo P (2018). Mitofusin 2: from functions to disease. Cell Death Dis..

[31] Qi Y (2016). Structures of human mitofusin 1 provide insight into mitochondrial tethering. J. Cell. Biol..

[32] Cao YL (2017). MFN1 structures reveal nucleotide-triggered dimerization critical for mitochondrial fusion. Nature.

[33] Yan L (2018). Structural basis for GTP hydrolysis and conformational change of MFN1 in mediating membrane fusion. Nat. Struct. Mol. Biol..

[34] Eura Y, Ishihara N, Yokota S, Mihara K (2003). Two mitofusin proteins, mammalian homologues of FZO, with distinct functions are both required for mitochondrial fusion. J. Biochem..

[35] Rocha AG (2018). MFN2 agonists reverse mitochondrial defects in preclinical models of Charcot-Marie-Tooth disease type 2A. Science.

[36] Chappie JS, Acharya S, Leonard M, Schmid SL, Dyda F (2010). G domain dimerization controls dynamin’s assembly-stimulated GTPase activity. Nature.

[37] Bian X (2011). Structures of the atlastin GTPase provide insight into homotypic fusion of endoplasmic reticulum membranes. Proc. Natl Acad. Sci. USA..

[38] Byrnes LJ, Sondermann H (2011). Structural basis for the nucleotide-dependent dimerization of the large G protein atlastin-1/SPG3A. Proc. Natl Acad. Sci. USA..

[39] El Fissi N. et al. Mitofusin gain and loss of function drive pathogenesis in Drosophila models of CMT2A neuropathy. *EMBO Rep*. **19**,8, (2018). 10.15252/embr.201745241 PMC6073211 29898954

[40] Sawyer SL (2015). Homozygous mutations in MFN2 cause multiple symmetric lipomatosis associated with neuropathy. Hum. Mol. Genet..

[41] Detmer SA, Chan DC (2007). Complementation between mouse Mfn1 and Mfn2 protects mitochondrial fusion defects caused by CMT2A disease mutations. J. Cell Biol..

[42] Chen Y (2017). Conformational dynamics of dynamin-like MxA revealed by single-molecule FRET. Nat. Commun..

[43] Low HH, Sachse C, Amos LA, Löwe J (2009). Structure of a bacterial dynamin-like protein lipid tube provides a mechanism for assembly and membrane curving. Cell.

[44] Liu J, Noel JK, Low HH (2018). Structural basis for membrane tethering by a bacterial dynamin-like pair. Nat. Commun..

[45] Kumar S (2016). Activation of mitofusin2 by Smad2-RIN1 complex during mitochondrial fusion. Mol. Cell.

[46] Ishihara N, Maeda M, Ban T, Mihara K (2017). Cell-free mitochondrial fusion assay detected by specific protease reaction revealed Ca2+ as regulator of mitofusin-dependent mitochondrial fusion. J. Biochem..

[47] Hunkeler M (2018). Structural basis for regulation of human acetyl-CoA carboxylase. Nature.

[48] Brandt T., Cavellini L., Kuhlbrandt W., Cohen M. M. A mitofusin-dependent docking ring complex triggers mitochondrial fusion in vitro. *eLife* **5**, e14618 (2016). 10.7554/eLife.14618 PMC4929004 27253069

[49] Hoppins S (2011). The soluble form of Bax regulates mitochondrial fusion via MFN2 homotypic complexes. Mol. Cell.

[50] Calvo J (2009). Genotype-phenotype correlations in Charcot-Marie-Tooth disease type 2 caused by mitofusin 2 mutations. Arch. Neurol..

[51] Liu TY (2012). Lipid interaction of the C terminus and association of the transmembrane segments facilitate atlastin-mediated homotypic endoplasmic reticulum fusion. Proc. Natl Acad. Sci. USA..

[52] Wang Q-S (2018). Upgrade of macromolecular crystallography beamline BL17U1 at SSRF. Nucl. Sci. Tech..

[53] Kabsch W (2010). Xds. Acta Crystallogr. D. Biol. Crystallogr..

[54] Vagin A, Teplyakov A (1997). *MOLREP*: an automated program for molecular replacement. J. Appl Cryst..

[55] Emsley P, Cowtan K (2004). Coot: model-building tools for molecular graphics. Acta Crystallogr. D. Biol. Crystallogr..

[56] Murshudov GN, Vagin AA, Dodson EJ (1997). Refinement of macromolecular structures by the maximum-likelihood method. Acta Crystallogr. D. Biol. Crystallogr..

[57] Adams PD (2010). PHENIX: a comprehensive python-based system for macromolecular structure solution. Acta Crystallogr. Sect. D., Biol. Crystallogr..

[58] Chen VB (2010). MolProbity: all-atom structure validation for macromolecular crystallography. Acta Crystallogr. D. Biol. Crystallogr..

[59] Laskowski RA, MacArthur MW, Moss DS, Thornton JM (1993). *PROCHECK*: a program to check the stereochemical quality of protein structures. J. Appl. Cryst..

[60] Madeira F (2019). The EMBL-EBI search and sequence analysis tools APIs in 2019. Nucleic Acids Res..

[61] Ashkenazy H (2016). ConSurf 2016: an improved methodology to estimate and visualize evolutionary conservation in macromolecules. Nucleic Acids Res..

