# Supplementary material for: Structural insights of human mitofusin-2 into mitochondrial fusion and CMT2A onset
Source: Nat Commun. 2019 Oct 29;10:4914. doi: 10.1038/s41467-019-12912-0 (PMC6820788; doi:10.1038/s41467-019-12912-0)
Supplement: Supplementary file 1 — Supplementary Information [file 41467_2019_12912_MOESM1_ESM.pdf]

Supplementary Information for

Structural insights of human mitofusin-2 into mitochondrial fusion and CMT2A onset

Yu-Jie Li[1], Yu-Lu Cao[1], Jian-Xiong Feng[1], Yuanbo Qi[2], Shuxia Meng[3], Jie-Feng Yang[1], Ya-Ting Zhong[1], Sisi Kang[4], Xiaoxue Chen[4], Lan Lan[5,6], Li Luo[1], Bing Yu[1], Shoudeng Chen[4], David C. Chan[3], Junjie Hu[2,5,6], Song Gao[1,7,*]

Corresponding author: Song Gao (gaosong@sysucc.org.cn). Yu-Jie Li, Yu-Lu Cao and Jian-Xiong Feng contributed equally to this work.

1, State Key Laboratory of Oncology in South China, Collaborative Innovation Center for Cancer Medicine, Sun Yat-sen University Cancer Center, Guangzhou 510060, China.

2, Department of Genetics and Cell Biology, College of Life Sciences, Nankai University, Tianjin 300071, China.

3, Division of Biology and Biological Engineering, California Institute of Technology, Pasadena, CA, USA.

4, Department of Experimental Medicine, Guangdong Provincial Key Laboratory of Biomedical Imaging, The Fifth affiliated Hospital, Sun Yat-sen University, Zhuhai 519000, China.

5, National Laboratory of Biomacromolecules, CAS Center for Excellence in Biomacromolecules, Institute of Biophysics, Chinese Academy of Sciences, Beijing 100101, China

6, University of Chinese Academy of Sciences, Beijing 100101, China

7, Guangzhou Regenerative Medicine and Health Guangdong Laboratory, Guangzhou 510530, China.

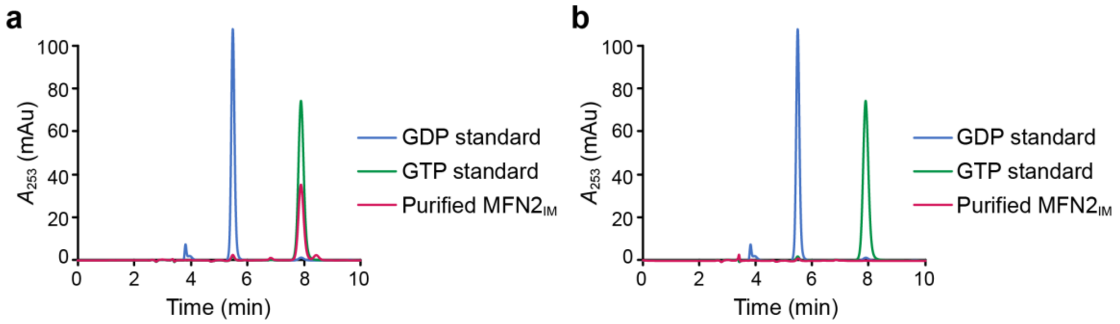

Supplementary Fig. 1 Nucleotide-loading states of MFN2IM after purification

a, HPLC chromatograph shows that freshly purified $MFN2_{IM}$ is loaded with GTP.

b, $MFN2_{IM}$ purified with optimized protocol is free of guanine nucleotides.

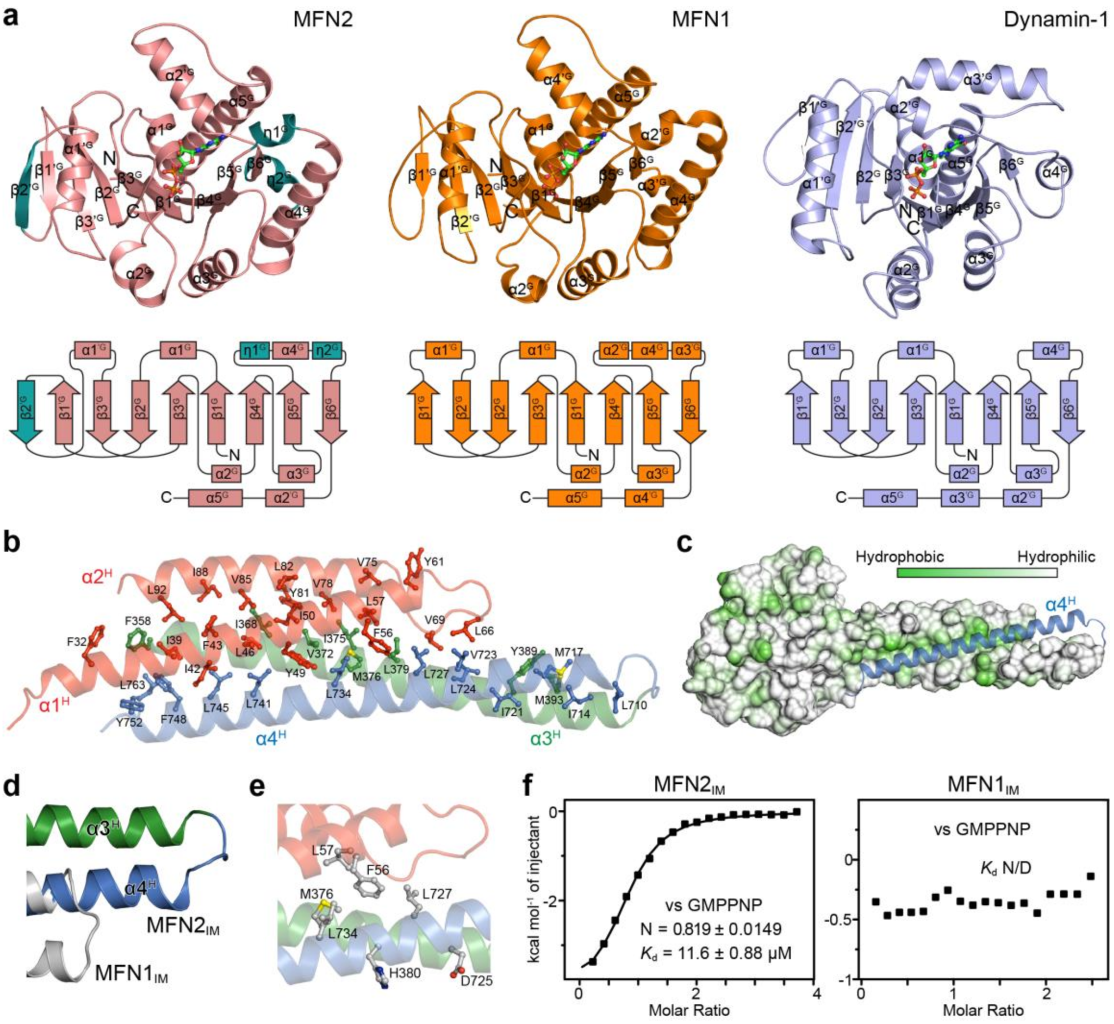

Supplementary Fig. 2 Biochemical and structural features of MFN2$_{IM}$

a, The structure and topology diagram of the G domains of MFN2$_{IM}$, MFN1$_{IM}$ (Protein Data Bank code 5GOM), and dynamin-1 (2X2E). Secondary structural elements are not drawn to scale. The MFN2-specific 3_{10} helices ($\eta1^G$ and $\eta2^G$) and $\beta2'^G$ are colored cyan. Note that because of extra secondary elements, element labeling of MFN2$_{IM}$ is slightly different from that of MFN1$_{IM}$ [1].

b, Hydrophobic network within HD1. Side chains of the residues involved in the network are shown in the same color as the helices they belong to.

c, The conventional HR2 is part of the HD1. $\alpha4^H$ comprising a large portion of HR2 is colored blue. and the rest part of MFN2$_{IM}$ is shown as hydrophobic surface representation. Note the extensive green patch underneath $\alpha4^H$, which entails a tight hydrophobic association.

d, Extended $\alpha3^H$ and $\alpha4^H$ of MFN2$_{IM}$ as compared to MFN1$_{IM}$. MFN1$_{IM}$ is colored grey. $\alpha3^H$ and $\alpha4^H$ of MFN2$_{IM}$ are colored green and blue, respectively.

e, Distribution of residues in HD1. No intramolecular interaction exists between Met376 and Leu727, or between His380 and Asp725.

f, Binding of GMPPNP with MFN2$_{IM}$ and MFN1$_{IM}$ measured by ITC. N/D, not determined.

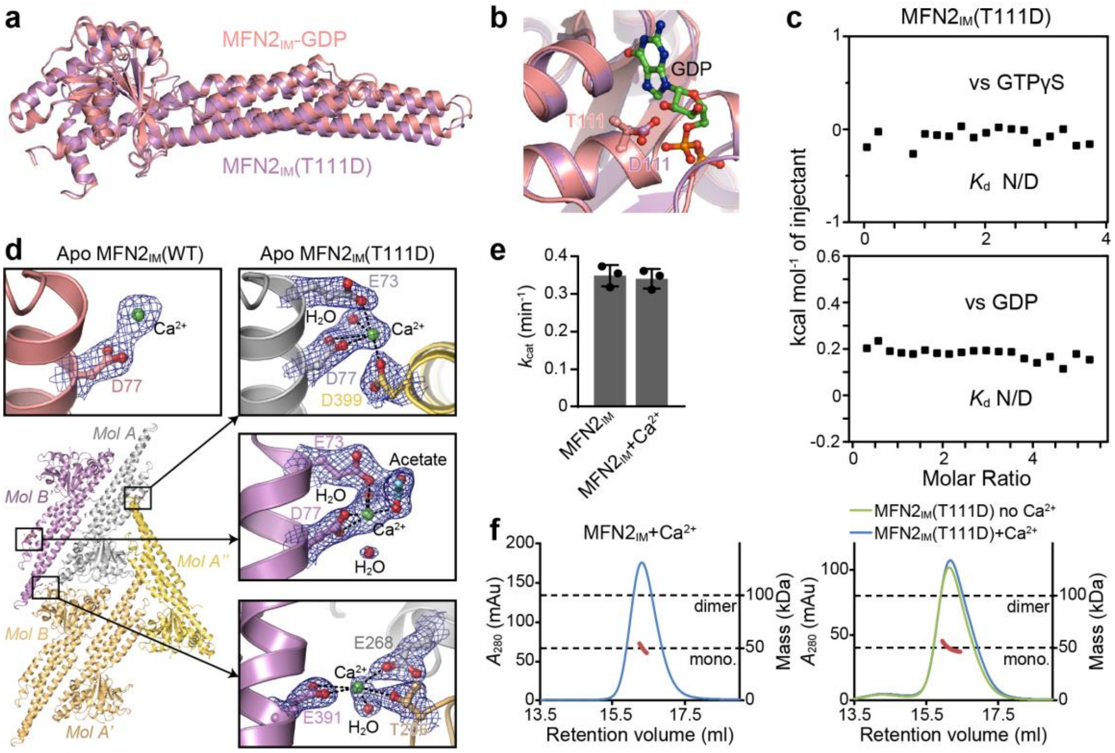

Supplementary Fig. 3 Structure of truncated MFN2$_{IM}$(T111D)

a, Structural comparison between MFN2$_{IM}$(T111D) (violet) and GDP-bound MFN2$_{IM}$ (pink).

b, Details of the T111D mutation. The GDP molecule is shown as ball-and-stick model. Note that the side chain of negatively-charged Asp111 causes a space- and charge-hindrance to the accommodation of the phosphate groups of GDP.

c, Binding of GTPγS and GDP with MFN2$_{IM}$(T111D) measured by ITC. N/D, not determined.

d, The coordination of Ca^{2+} of apo MFN2$_{IM}$ (upper-left) and MFN2$_{IM}$(T111D) (right). Involved residues and acetate ion and are shown as ball-and-stick models. Electron density map is shown as blue mesh at a contour level of 1.0σ.

e, GTP turnover rates of wild-type MFN2$_{IM}$ in the absence and presence of 10 mM $CaCl_2$. Error bars indicate s.d. (n = 3). Source data are provided as a Source Data file.

f, Dimerization property of nucleotide-free wild-type MFN2$_{IM}$ (left) and MFN2$_{IM}$(T111D) (right) after incubated with 10 mM $CaCl_2$ for 2 h. 5 mM $CaCl_2$ were supplied to the running buffer of the RALS assay.

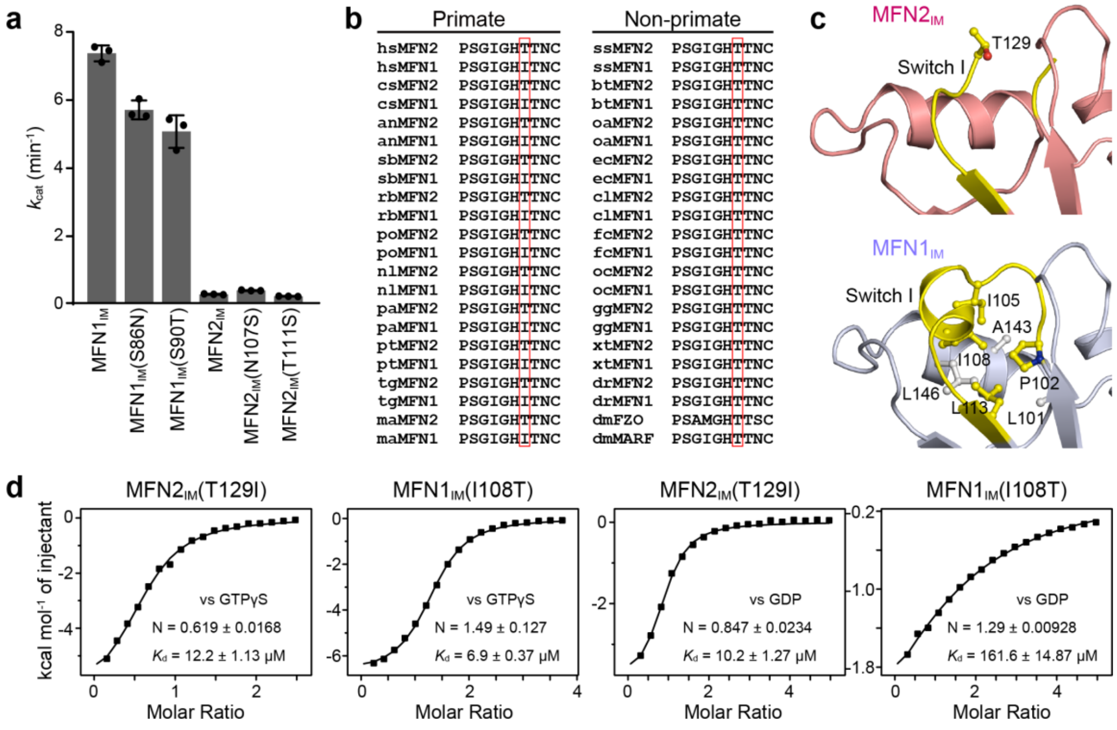

Supplementary Fig. 4 The difference in the switch I of human mitofusins

a, GTP turnover rates of wild-type $MFN1_{IM}$/$MFN2_{IM}$ and corresponding mutants. Error bars indicate s.d. (n = 3). Source data are provided as a Source Data file.

b, Sequence alignment of primate and non-primate mitofusins in switch I. The equivalent positions for Thr129 of human MFN2 are highlighted by red frames. Primates: hs, *Homo sapiens*; cs, *Chlorocebus sabaeus*; an, *Aotus nancymaae*; sb, *Saimiri boliviensis*; rb, *Rhinopithecus bieti*; po, *Pongo abelii*; nl, *Nomascus leucogenys*; pa, *Papio anubis*; pt, *Piliocolobus tephrosceles*; tg, *Theropithecus gelada*; ma, *Macaca mulatta*. Non-primates: ss, *Sus scrofa*; bt, *Bos taurus*; oa, *Ovis aries*; ec, *Equus caballus*; cl, *Canis lupus familiaris*; fc, *Felis catus*; oc, *Oryctolagus cuniculus*; gg, *Gallus gallus*; xt, *Xenopus tropicalis*; dr, *Danio rerio*; dm, *Drosophila melanogaster*.

c, Comparison of the configuration of switch I between $MFN2_{IM}$ and $MFN1_{IM}$ (PDB code 5GO4) in the nucleotide-free state. Switch I regions are highlighted in yellow. Thr129 of $MFN2_{IM}$, Ile108 and other residues involved in the hydrophobic network of $MFN1_{IM}$ are shown as stick-and-ball models.

d, Binding of GTPγS and GDP with $MFN2_{IM}$(T129I) and $MFN1_{IM}$(I108T).

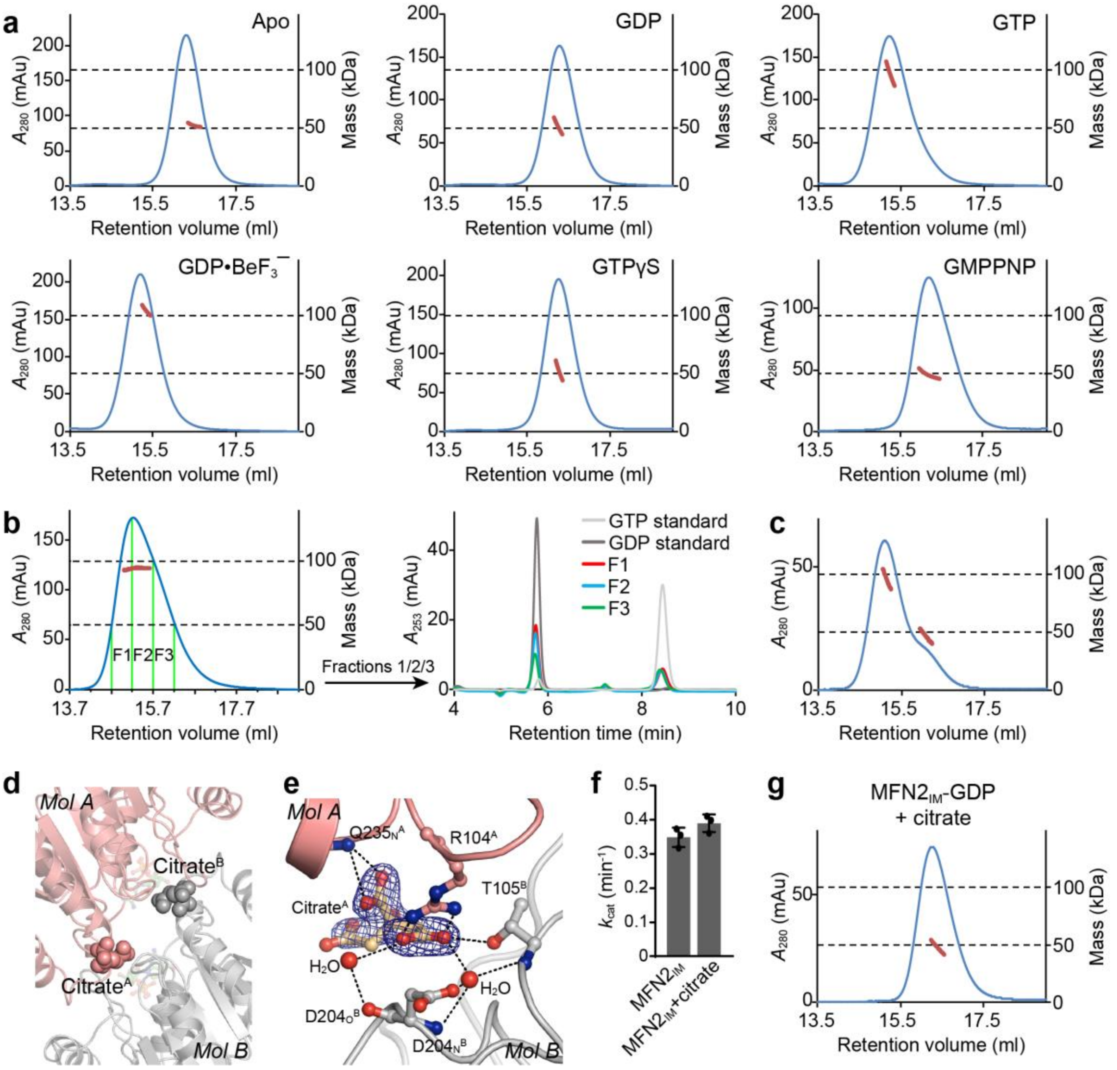

Supplementary Fig. 5 Dimerization test of $MFN2_{IM}$

a, Dimerization property of $MFN2_{IM}$ in different nucleotide-loading states.

b, GTP-induced $MFN2_{IM}$ dimers slowly hydrolyze GTP while keeping associated. Left: RALS analysis of $MFN2_{IM}$-GTP mixture after incubation for 2 h. Right: HPLC analysis of the nucleotides in different fractions of the dimer peak in gel-filtration.

c, Dimerization property of freshly purified GTP-loaded $MFN2_{IM}$.

d, Location of the citrate ions (shown as spheres) at the G interface.

e, Interactions of citrate with $MFN2_{IM}$. The citrate ion and corresponding residues are shown as ball-and-stick models. Electron density map for citrate is shown as blue mesh at a contour level of 1.2σ.

f, GTP turnover rates of $MFN2_{IM}$ in the absence and presence of 10 mM sodium citrate. Error bars indicate s.d. (n = 3). Source data are provided as a Source Data file.

g, Dimerization property of $MFN2_{IM}$ in the presence of GDP after incubated with 10 mM sodium citrate for 2 h. 5 mM sodium citrate were supplied to the running buffer of the RALS assay.

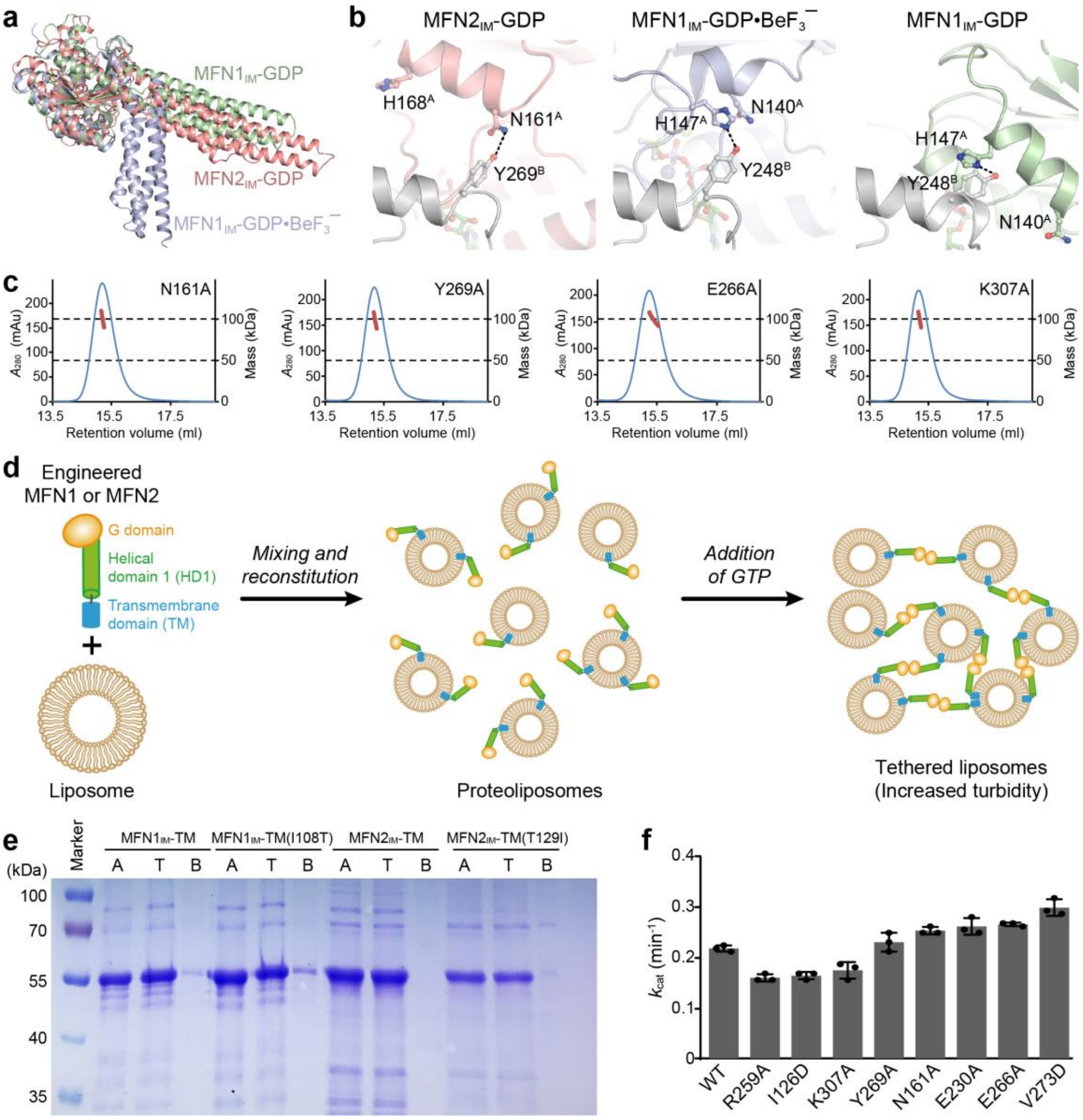

Supplementary Fig. 6 The differences of the MFN2$_{IM}$ and MFN1$_{IM}$ dimers

a, Structural superposition of the G domains of the MFN1$_{IM}$ in the post-transition state, PDB code 5GOM), MFN1$_{IM}$ in the transition state (PDB code 5YEW) and MFN2$_{IM}$ in the GDP-bound state. Note that the relative position of HD1 of MFN2$_{IM}$ is in between those of the two MFN1$_{IM}$.

b, Different residue contacts of the MFN2$_{IM}$-GDP dimer, MFN1$_{IM}$-GDP•BeF_3^- dimer (PDB code 5YEW) and MFN1$_{IM}$-GDP dimer (PDB code 5GOM). Corresponding residues are shown as ball-and-stick models.

c, Dimerization property of MFN2$_{IM}$ G interface mutants in the presence of GDP•BeF_3^-.

d, Experimental process of spectroscopic liposome tethering assay.

e, Purified and reconstituted TM-containing MFN1$_{IM}$ and MFN2$_{IM}$ were floated in a sucrose gradient. Top (T) and bottom (B) fractions were analyzed by SDS/PAGE and Coomassie blue staining. “A” denotes total proliposomes. Source data are provided as a Source Data file.

f, GTP turnover rates of wild-type MFN2$_{IM}$ and the G interface mutants. Error bars indicate s.d. (n = 3). Source data are provided as a Source Data file.

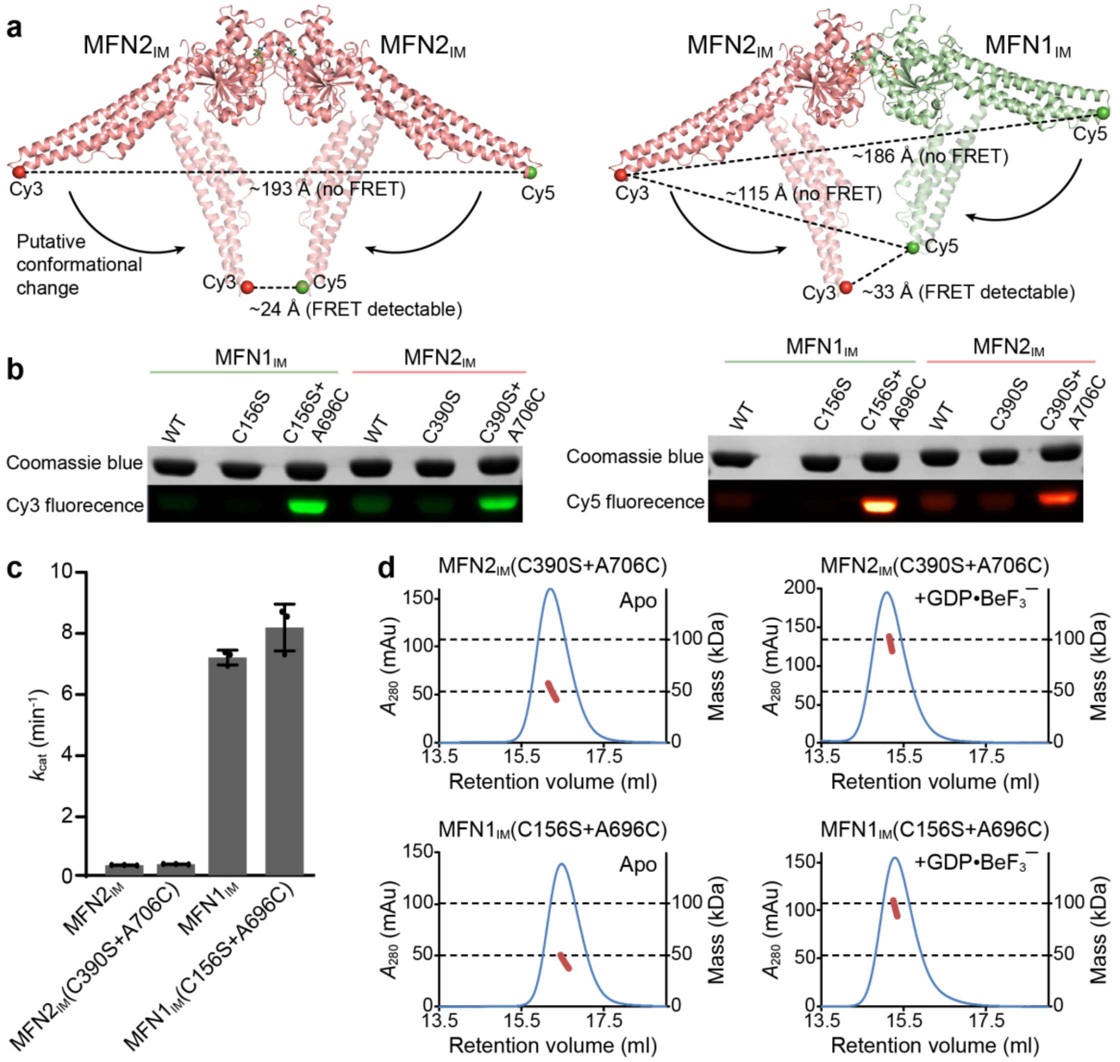

Supplementary Fig. 7 The design and quality control of the FRET assay

a, The selected $MFN1_{IM}$/$MFN2_{IM}$ sites of fluorescent labeling for the FRET assay, as indicated by red (Cy3) or green (Cy5) spheres. The distances between the fluorescence labels were estimated based on the crystal structures of $MFN2_{IM}$-GDP and $MFN1_{IM}$-GDP•BeF_3^- (PDB code 5YEW). The FRET signals can be detected only when associated $MFN2_{IM}$ and $MFN1_{IM}$ both change to the closed conformation.

b, Site-specific dye-labeling of engineered $MFN1_{IM}$ and $MFN2_{IM}$. The SDS-PAGE results show that altering exposed cysteines to serines (C156S for $MFN1_{IM}$ and C390S for $MFN2_{IM}$) has successfully prevented non-specific fluorescent labeling. The introduction of designated mutations, namely A696C for $MFN1_{IM}$(C156S) and A706C for $MFN2_{IM}$(C390S), allows efficient labeling by fluorescent dyes. Source data are provided as a Source Data file.

c, GTP turnover rates of $MFN2_{IM}$ and $MFN1_{IM}$ constructs used for FRET assays. Error bars indicate s.d. (n = 3). GTPase activity is not affected by introduced mutations. Source data are provided as a Source Data file.

d, Dimerization property of $MFN2_{IM}$ and $MFN1_{IM}$ constructs used for FRET assays in the absence and presence of GDP•BeF_3^-. G interface-dependent dimerization is not affected by introduced mutations.

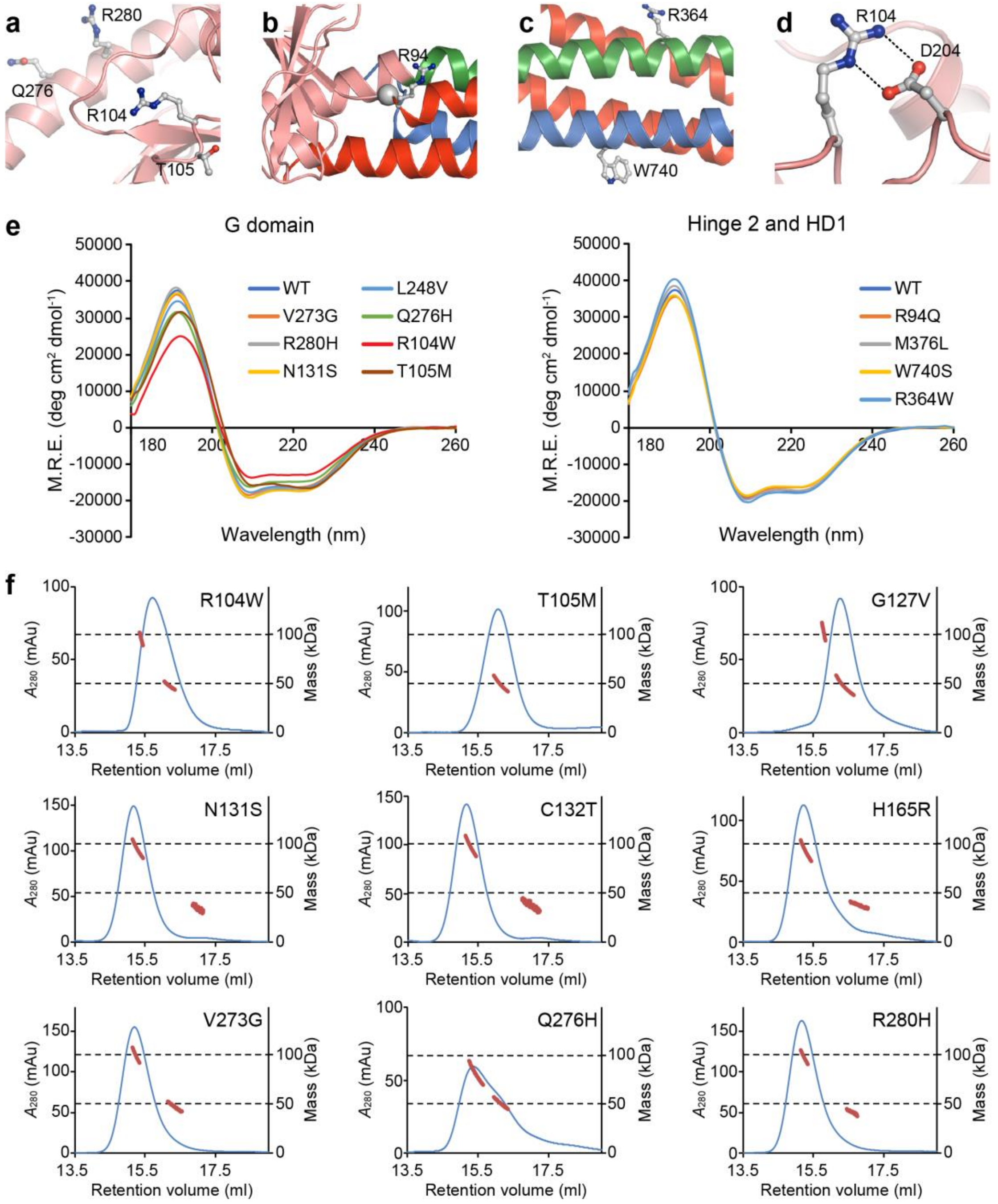

Supplementary Fig. 8 Structural features of CMT2A-related mutants

a-d, CMT2A-related mutation sites on the surface of MFN2.

e, CD spectra of wild-type $MFN2_{IM}$ and CMT2A-related mutants. M.R.E. denotes mean residue epllipticity.

f, Dimerization property of CMT2A-mutants at the G interface in the presence of GDP•BeF_3^-.

Supplementary Table 1 Summary of CMT2A-related mutations

Mutations	Zone*	Con.**	E/B***	G/A****	Interaction MFN1/2*****	Mutations	Zone*	Con.**	E/B***	G/A****	Interaction MFN1/2*****
V69F[2]	V	S	B	—	—	R259L[3]	I/II	I	E	—	—
L76P[2]	V	S	E	H	—	S263P[4, 5]	II	I	E	—	—
L92R[6]	IV	I	B	—	—	V273G[7]	II	I	E	C	+++/++
L92P[5, 8, 9]	IV	I	B	—	—	Q276R[10]	II	I	E	—	—
R94G[11]	III	I	E	—	—	Q276H[12, 13, 14]	II	I	E	C	+++/+
R94Q[2, 11, 13, 14. 15. 16. 17. 18]	III	I	E	H	+++/+++	H277R[9]	II	I	E	—	—
R94W[4, 8, 10, 11. 13. 17. 19]	III	I	E	H	+++/+++	H277Y[20]	II	I	E	—	—
R104W[21, 22, 23. 24. 25]	I/II	I	E	L	-/+	R280H[2, 8, 26]	II	I	E	H	+++/++
T105M[7, 11]	I/II	I	E	H	-/-	F284Y[18]	V	I	E	—	—
P123L[9]	I	I	E	—	—	E288D[27]	V	I	E	—	—
G127V[28]	II	I	E	L	-/++	G298R[13]	V	U	E	—	—
G127D[5]	II	I	E	—	—	E347V[28]	V	I	E	—	—
H128R[20]	I	I	E	—	—	S350P[4, 8]	V	I	E	—	—
N131S[29]	I	I	E	H	+++/+++	T356A[30]	IV	I	E	—	—
C132T[15]	V	I	E	L	+++/+++	K357N[18]	III	I	E	—	—
L146F[31]	V	I	B	—	—	H361Y[10, 11]	IV	I	E	—	—
S156I[20]	V	I	E	—	—	T362M[5, 32, 33]	IV	I	E	—	—
A164V[33]	V	I	E	—	—	R364W[8, 10, 11. 19. 34]	IV	I	E	H	+++/+
H165L[35]	II	I	E	—	—	R364Q[13, 14, 20]	IV	I	E	—	—
H165R[4, 5, 8, 9]	II	I	E	H	+++/++	R364P[11, 20, 36]	IV	I	E	H	+++/+
H165Y[9]	II	I	E	—	—	M376L[6]	V	I	B	C	+++/+++
I203M[13]	V	N	B	—	—	M376I[9, 28]	V	I	B	—	—
T206I[10]	V	I	E	—	—	M376V[13]	V	I	B	—	—
D210V[37]	V	I	E	—	—	M376T[5]	V	I	B	—	—
I213T[7]	V	I	B	—	—	S378P[25]	V	I	E	—	—
D214N[33]	V	I	E	—	—	Q386P[9]	V	U	E	—	—
F216S[32]	V	I	E	—	—	C390F[11]	V	U	E	—	—
L218P[38]	V	I	E	H	+++/++	C390R[33]	V	U	E	—	—
F223Y[39]	V	I	B	—	—	R400P[23]	V	I	E	—	—
F223L[18]	V	I	B	—	—	R707W[17, 33]	V	U	E	—	—
L233V[34]	V	I	E	—	—	L710P[9]	V	I	E	—	—
T236M[18]	II	I	E	—	—	A716T[11, 17]	V	U	E	—	—
V244M[18]	V	I	B	—	—	L724P[40]	V	I	B	—	—
L248V[11]	IV	I	E	H	+++/+++	A738V[40]	V	I	E	—	—
S249F[24]	IV	I	E	—	—	W740S[2, 11]	V	U	E	C	+++/+++
R250W[9]	IV	S	E	—	—	W740R[19]	V	U	E	—	—
R250Q[6, 9]	IV	S	E	—	—	W740C[20]	V	U	E	—	—
P251A[2]	IV	I	E	—	—	E744M[34]	V	I	E	—	—
P251R[6, 11]	IV	I	E	L	++/++	L745P[20]	V	I	B	—	—
P251L[41]	IV	I	E	H	+++/++	M747T[20]	V	U	E	—	—
N252K[13]	IV	I	E	—	—	H750P[11]	V	U	E	—	—

*Zone: I, nucleotide-binding site; II, G interface; III, hinge 2; IV, G domain-HD1 interface; V, other area.

**Conservation between human MFN1 and MFN2: I, identical; S, similar; U, unconserved.

***Exposed (E) or buried (B).

****GTPase activity (G/A) compared to wild-type: H, (statistically) higher; C, comparable; L, lower. Significance was calculated with Student's *t*-test (n = 3). Cut-off value: $p < 0.05$.

*****Interaction with MFN1/MFN2: -, no interaction; +/++/+++, moderate/medium/strong interaction.

Supplementary Table 2 List of primers

	Primers	Sequence 5’-3’
1	pET-28_MFN2$_{IM}$-FW	GGCGGATCCGCTGAGGTGAATGCATCCCCAC
2	pET-28_MFN2$_{IM}$-RE	GGCCTCGAGCTATCTGCTGGGCTGCAGGTAC
3	MFN2$_{IM}$-overlap-FW	GAGCGGCAAGACCGAACCCGGGAGAACCTGGAGCAGG
4	MFN2$_{IM}$-overlap-RE	CAGGTTCTCCCGGGTTCGGTCTTGCCGCTCTTCACGC
5	MBP-tagged MFN1$_{IM}$-FW	ATTTGCGGCCGCAGCTGAGGTGAATGCATCCCC
6	MBP-tagged MFN1$_{IM}$-RE	CCGCTCGAGCTATCTGCTGGGCTGCAGGTAC
7	MBP-tagged MFN2$_{IM}$-FW	GGTTCTGTCGACTCTGCGGCCGCAGCTGAGGTGAATGCATCCCCAC
8	MBP-tagged MFN2$_{IM}$-RE	GTGGTGGTGGTGGTGCTCGAGCTATCTGCTGGGCTGCAGGTAC
9	PGEX-6P-3_MFN2$_{IM}$-FW	CGCGGATCCATGGCTGAGGTGAATGCATCCCCACTTAA
10	MFN2$_{IM}$-TM-RE1	ACCAGAATACCCATGCTAGCGCTCGCAGCCGATCGGTCTTGCCG
11	MFN2$_{IM}$-TM-FW1	CGGCAAGACCGATCGGCTGCGAGCGCTAGCATGGGTATTCTGGT
12	MFN2$_{IM}$-TM-RE2	GGTTCTCCCGGGTGGCTGCGGATGCGTAAACATACAGCAGG
13	MFN2$_{IM}$-TM-FW2	CCTGCTGTATGTTTACGCATCCGCAGCCACCCGGGAGAACC
14	PGEX-6P-3_MFN2$_{IM}$-RE	CCGCTCGAGTTATCTGCTGGGCTGCAG
15	PGEX-6P-3_MFN1$_{IM}$-FW	CGCGGATCCATGGATTACAAGGATGACGACGATAAGGCCGAA
16	MFN1$_{IM}$-TM-RE1	ACGATGATGATGCCCATGCTACTGCCGCCACTGCCACTGCCACTGCC
17	MFN1$_{IM}$-TM-FW1	GGCAGTGGCAGTGGCAGTGGCGGCAGTAGCATGGGCATCATCATCGT
18	MFN1$_{IM}$-TM-RE2	TGCGATACTTCCGGAACCGTACAGGTACAGTGCACCAT
19	MFN1$_{IM}$-TM-FW2	GGTGCACTGTACCTGTACGGTTCCGGAAGTATCGCA
20	PGEX-6P-3_MFN1$_{IM}$-RE	CCGCTCGAGTTAAGCGTAATCTGGAACATCGTATGGGTAGCTTTCTTCGTT
21	PGEX-6P-1_MFN2$_{IM}$-FW	GGCGGATCCGCTGAGGTGAATGCATCCCCAC
22	MFN2$_{IM}$-TM^{Sac1}-FW1	GCCTTGACCGTTTTAGGTGCTACGATATTTTTTCCTAAAGATAGATTTACCAGCAGTAAGACCCGGGAGAACCTGGAGCAG
23	MFN2$_{IM}$-TM^{Sac1}-RE1	CGTAGCACCTAAAACGGTCAAGGCTGCGCAAATGATCATTGGGATCAGCTGAATATATCGGTCTTGCCGCTCTTCACGC
24	MFN2$_{IM}$-TM^{Sac1}-FW2	CGATTGTCTTGGCGCTTTCAACCAAATTCATGTTTAAGAACGGTATTCAGTTTGTCACCCGGGAGAACCTGGAGCAG
25	MFN2$_{IM}$-TM^{Sac1}-RE2	GGTTGAAAGCGCCAAGACAATCGACGCACCTGCAAAATACAGCAAATTCTTACTGCTGGTAAATCTATCTTTAGG
26	PGEX-6P-1_MFN2$_{IM}$-RE	GGCCTCGAGCTATCTGCTGGGCTGCAGGTAC
27	PGEX-6P-1_MFN1$_{IM}$-FW	GGCGGATCCATGGCAGAACCTGTTTCTCCACTG
28	MFN1$_{IM}$-TM^{Sac1}-FW1	GCCTTGACCGTTTTAGGTGCTACGATATTTTTTCCTAAAGATAGATTTACCAGCAGTAAGGCTAGATTACCCAAAGAAATAGATCAG
29	MFN1$_{IM}$-TM^{Sac1}-RE1	CGTAGCACCTAAAACGGTCAAGGCTGCGCAAATGATCATTGGGATCAGCTGAATATATGAATAATGCCTTTTATCTTCAGCTG
30	MFN1$_{IM}$-TM^{Sac1}-FW2	CGATTGTCTTGGCGCTTTCAACCAAATTCATGTTTAAGAACGGTATTCAGTTTGTCGCTAGATTACCCAAAGAAATAGATCAG
31	MFN1$_{IM}$-TM^{Sac1}-RE2	GGTTGAAAGCGCCAAGACAATCGACGCACCTGCAAAATACAGCAAATTCTTACTGCTGGTAAATCTATCTTTAGG
32	PGEX-6P-1_MFN1$_{IM}$-RE	GGCCTCGAGTTAGGATTCTTCATTGCTTGAAGGTAG
33	pQCXIP_MFN2(I126D)-FW	ATCCACGCTGTTTTGACCTC
34	pQCXIP_MFN2(I126D)-RE	CTGTGCCCCCAACCCGCAGGAAGCAATTGGTGGTATGACCATCC CCAGATGGCAG
35	pQCXIP_MFN2-FW	GGGAAGAGCACCGTGATCAA
36	MFN2(N161A)-FW	TGGGCCAGTTGGGCCACAGTCTTGAC
37	MFN2(N161A)-RE	GTCAAGACTGTGGCCCAACTGGCCCA
38	pQCXIP_MFN2-RE	AGCAGCGGTCAGACAGGTT
39	MFN2(K307A)-FW	CTGAGAACCTCCGCGGCAGACACGAA
40	MFN2(K307A)-RE	TTCGTGTCTGCCGCGGAGGTTCTCAG

Supplementary References

1. Cao YL, Meng S, Chen Y, Feng JX, Gu DD, Yu B*, et al.* MFN1 structures reveal nucleotide-triggered dimerization critical for mitochondrial fusion. *Nature* 2017, **542**(7641)**:** 372-376.

2. Züchner S, Mersiyanova IV, Muglia M, Bissar-Tadmouri N, Rochelle J, Dadali EL*, et al.* Mutations in the mitochondrial GTPase mitofusin 2 cause Charcot-Marie-Tooth neuropathy type 2A. *Nature genetics* 2004, **36**(5)**:** 449-451.

3. Ajroud-Driss S, Fecto F, Ajroud K, Yang Y, Donkervoort S, Siddique N*, et al.* A novel de novo MFN2 mutation causing CMT2A with upper motor neuron signs. *Neurogenetics* 2009, **10**(4)**:** 359-361.

4. Cho HJ, Sung DH, Kim BJ, Ki CS. Mitochondrial GTPase mitofusin 2 mutations in Korean patients with Charcot-Marie-Tooth neuropathy type 2. *Clinical genetics* 2007, **71**(3)**:** 267-272.

5. Chung KW, Kim SB, Park KD, Choi KG, Lee JH, Eun HW*, et al.* Early onset severe and late-onset mild Charcot-Marie-Tooth disease with mitofusin 2 (MFN2) mutations. *Brain : a journal of neurology* 2006, **129**(Pt 8)**:** 2103-2118.

6. McCorquodale DS, 3rd, Montenegro G, Peguero A, Carlson N, Speziani F, Price J*, et al.* Mutation screening of mitofusin 2 in Charcot-Marie-Tooth disease type 2. *Journal of neurology* 2011, **258**(7)**:** 1234-1239.

7. Lawson VH, Graham BV, Flanigan KM. Clinical and electrophysiologic features of CMT2A with mutations in the mitofusin 2 gene. *Neurology* 2005, **65**(2)**:** 197-204.

8. Chung KW, Suh BC, Cho SY, Choi SK, Kang SH, Yoo JH*, et al.* Early-onset Charcot-Marie-Tooth patients with mitofusin 2 mutations and brain involvement. *Journal of neurology, neurosurgery, and psychiatry* 2010, **81**(11)**:** 1203-1206.

9. Verhoeven K, Claeys KG, Zuchner S, Schroder JM, Weis J, Ceuterick C*, et al.* MFN2 mutation distribution and genotype/phenotype correlation in Charcot-Marie-Tooth type 2. *Brain : a journal of neurology* 2006, **129**(Pt 8)**:** 2093-2102.

10. Zuchner S, De Jonghe P, Jordanova A, Claeys KG, Guergueltcheva V, Cherninkova S*, et al.* Axonal neuropathy with optic atrophy is caused by mutations in mitofusin 2. *Annals of neurology* 2006, **59**(2)**:** 276-281.

11. Feely SM, Laura M, Siskind CE, Sottile S, Davis M, Gibbons VS*, et al.* MFN2 mutations cause severe phenotypes in most patients with CMT2A. *Neurology* 2011, **76**(20)**:** 1690-1696.

12. Ching CK, Lau KK, Yu KW, Chan YW, Mak MC. A novel mitofusin 2 gene mutation causing

Charcot-Marie-Tooth type 2A disease in a Chinese family. *Chinese medical journal* 2010, **123**(11)**:** 1466-1469.

13. Casasnovas C, Banchs I, Cassereau J, Gueguen N, Chevrollier A, Martinez-Matos JA*, et al.* Phenotypic spectrum of MFN2 mutations in the Spanish population. *Journal of medical genetics* 2010, **47**(4)**:** 249-256.

14. Banchs I, Casasnovas C, Montero J, Martinez-Matos JA, Volpini V. Two Spanish families with Charcot-Marie-Tooth type 2A: clinical, electrophysiological and molecular findings. *Neuromuscular disorders : NMD* 2008, **18**(12)**:** 974-978.

15. Zhang RX, Fu M, Zi XH, Li XB, Zhang FF, Xia K*, et al.* [Mutation analysis of MFN2 gene in Chinese patients with Charcot-Marie-Tooth disease]. *Zhonghua yi xue za zhi* 2009, **89**(47)**:** 3324-3327.

16. Neusch C, Senderek J, Eggermann T, Elolff E, Bahr M, Schneider-Gold C. Mitofusin 2 gene mutation (R94Q) causing severe early-onset axonal polyneuropathy (CMT2A). *European journal of neurology* 2007, **14**(5)**:** 575-577.

17. Braathen GJ, Sand JC, Lobato A, Hoyer H, Russell MB. MFN2 point mutations occur in 3.4% of Charcot-Marie-Tooth families. An investigation of 232 Norwegian CMT families. *BMC medical genetics* 2010, **11:** 48.

18. Kijima K, Numakura C, Izumino H, Umetsu K, Nezu A, Shiiki T*, et al.* Mitochondrial GTPase mitofusin 2 mutation in Charcot-Marie-Tooth neuropathy type 2A. *Human genetics* 2005, **116**(1-2)**:** 23-27.

19. Lv H, Wang L, Li W, Qiao X, Li Y, Wang Z*, et al.* Mitofusin 2 gene mutation causing early-onset CMT2A with different progressive courses. *Clinical neuropathology* 2013, **32**(1)**:** 16-23.

20. Calvo J, Funalot B, Ouvrier RA, Lazaro L, Toutain A, De Mas P*, et al.* Genotype-phenotype correlations in Charcot-Marie-Tooth disease type 2 caused by mitofusin 2 mutations. *Archives of neurology* 2009, **66**(12)**:** 1511-1516.

21. Del Bo R, Moggio M, Rango M, Bonato S, D'Angelo MG, Ghezzi S*, et al.* Mutated mitofusin 2 presents with intrafamilial variability and brain mitochondrial dysfunction. *Neurology* 2008, **71**(24)**:** 1959-1966.

22. Genari AB, Borghetti VH, Gouvea SP, Bueno KC, dos Santos PL, dos Santos AC*, et al.* Characterizing the phenotypic manifestations of MFN2 R104W mutation in Charcot-Marie-Tooth type 2. *Neuromuscular disorders : NMD* 2011, **21**(6)**:** 428-432.

23. Baets J, Deconinck T, De Vriendt E, Zimon M, Yperzeele L, Van Hoorenbeeck K*, et al.* Genetic spectrum of hereditary neuropathies with onset in the first year of life. *Brain : a journal of*

neurology 2011, **134**(Pt 9)**:** 2664-2676.

24. Abe A, Numakura C, Kijima K, Hayashi M, Hashimoto T, Hayasaka K. Molecular diagnosis and clinical onset of Charcot-Marie-Tooth disease in Japan. *Journal of human genetics* 2011, **56**(5)**:** 364-368.

25. Brockmann K, Dreha-Kulaczewski S, Dechent P, Bonnemann C, Helms G, Kyllerman M*, et al.* Cerebral involvement in axonal Charcot-Marie-Tooth neuropathy caused by mitofusin2 mutations. *Journal of neurology* 2008, **255**(7)**:** 1049-1058.

26. Vinci P, Lapi G. Anesthetic management in Charcot-Marie-Tooth disease type 2 due to a mutation in the mitofusin-2 gene. *Journal of anaesthesiology, clinical pharmacology* 2011, **27**(2)**:** 286-287.

27. Bergamin G, Dalla Torre C, Cacciavillani M, Lucchetta M, Boaretto F, Campagnolo M*, et al.* Novel mutation of the mitofusin 2 gene in a family with Charcot-Marie-Tooth disease type 2. *Muscle & nerve* 2014, **49**(1)**:** 145-146.

28. Engelfried K, Vorgerd M, Hagedorn M, Haas G, Gilles J, Epplen JT*, et al.* Charcot-Marie-Tooth neuropathy type 2A: novel mutations in the mitofusin 2 gene (MFN2). *BMC medical genetics* 2006, **7:** 53.

29. Fischer C, Trajanoski S, Papic L, Windpassinger C, Bernert G, Freilinger M*, et al.* SNP array-based whole genome homozygosity mapping as the first step to a molecular diagnosis in patients with Charcot-Marie-Tooth disease. *Journal of neurology* 2012, **259**(3)**:** 515-523.

30. Wang YW, Han WT, Jiang M, Lu CX, Li XF, Zhang X*, et al.* A novel mutation of the MFN2 gene in a Chinese family with Charcot-Marie-Tooth disease. *Genetics and molecular research : GMR* 2012, **11**(2)**:** 1454-1459.

31. Klein CJ, Kimmel GW, Pittock SJ, Engelstad JE, Cunningham JM, Wu Y*, et al.* Large kindred evaluation of mitofusin 2 novel mutation, extremes of neurologic presentations, and preserved nerve mitochondria. *Archives of neurology* 2011, **68**(10)**:** 1295-1302.

32. Polke JM, Laura M, Pareyson D, Taroni F, Milani M, Bergamin G*, et al.* Recessive axonal Charcot-Marie-Tooth disease due to compound heterozygous mitofusin 2 mutations. *Neurology* 2011, **77**(2)**:** 168-173.

33. Nicholson GA, Magdelaine C, Zhu D, Grew S, Ryan MM, Sturtz F*, et al.* Severe early-onset axonal neuropathy with homozygous and compound heterozygous MFN2 mutations. *Neurology* 2008, **70**(19)**:** 1678-1681.

34. Lin KP, Soong BW, Yang CC, Huang LW, Chang MH, Lee IH*, et al.* The mutational spectrum in a cohort of Charcot-Marie-Tooth disease type 2 among the Han Chinese in Taiwan. *PloS one*

2011, **6**(12)**:** e29393.

35. Marchesi C, Ciano C, Salsano E, Nanetti L, Milani M, Gellera C*, et al.* Co-occurrence of amyotrophic lateral sclerosis and Charcot-Marie-Tooth disease type 2A in a patient with a novel mutation in the mitofusin-2 gene. *Neuromuscular disorders : NMD* 2011, **21**(2)**:** 129-131.

36. Takahashi R, Ikeda T, Hamaguchi A, Iwasa K, Yamada M. Coenzyme Q10 therapy in hereditary motor sensory neuropathy type VI with novel mitofusin 2 mutation. *Internal medicine* 2012, **51**(7)**:** 791-793.

37. Rouzier C, Bannwarth S, Chaussenot A, Chevrollier A, Verschueren A, Bonello-Palot N*, et al.* The MFN2 gene is responsible for mitochondrial DNA instability and optic atrophy 'plus' phenotype. *Brain : a journal of neurology* 2012, **135**(Pt 1)**:** 23-34.

38. Ostern R, Fagerheim T, Hjellnes H, Nygard B, Mellgren SI, Nilssen O. Diagnostic laboratory testing for Charcot Marie Tooth disease (CMT): the spectrum of gene defects in Norwegian patients with CMT and its implications for future genetic test strategies. *BMC medical genetics* 2013, **14:** 94.

39. Park SY, Kim SY, Hong YH, Cho SI, Seong MW, Park SS. A novel double mutation in cis in MFN2 causes Charcot-Marie-Tooth neuropathy type 2A. *Neurogenetics* 2012, **13**(3)**:** 275-280.

40. Luigetti M, Fabrizi GM, Taioli F, Conte A, Del Grande A, Sabatelli M. Clinical, electrophysiological and pathological findings of a patient with CMT2 due to the p.Ala738Val mitofusin 2 mutation. *Journal of the neurological sciences* 2011, **307**(1-2)**:** 168-170.

41. Sivera R, Sevilla T, Vilchez JJ, Martinez-Rubio D, Chumillas MJ, Vazquez JF*, et al.* Charcot-Marie-Tooth disease: genetic and clinical spectrum in a Spanish clinical series. *Neurology* 2013, **81**(18)**:** 1617-1625.
