# Supplementary material for: Structural insights of human mitofusin-2 into mitochondrial fusion and CMT2A onset
Source: Nat Commun. 2019 Oct 29;10:4914. doi: 10.1038/s41467-019-12912-0 (PMC6820788; doi:10.1038/s41467-019-12912-0)
Supplement: Supplementary file 2 — Reporting Summary [file 41467_2019_12912_MOESM2_ESM.pdf]

nature research

Corresponding author(s): Song Gao

Last updated by author(s): Sep 30, 2019

Reporting Summary

Nature Research wishes to improve the reproducibility of the work that we publish. This form provides structure for consistency and transparency in reporting. For further information on Nature Research policies, see Authors & Referees and the Editorial Policy Checklist .

Statistics

For all statistical analyses, confirm that the following items are present in the figure legend, table legend, main text, or Methods section.

n/a	Confirmed	
☐	☒	The exact sample size (*n*) for each experimental group/condition, given as a discrete number and unit of measurement
☐	☒	A statement on whether measurements were taken from distinct samples or whether the same sample was measured repeatedly
☐	☒	The statistical test(s) used AND whether they are one- or two-sided<br>*Only common tests should be described solely by name; describe more complex techniques in the Methods section.*
☒	☐	A description of all covariates tested
☒	☐	A description of any assumptions or corrections, such as tests of normality and adjustment for multiple comparisons
☒	☐	A full description of the statistical parameters including central tendency (e.g. means) or other basic estimates (e.g. regression coefficient) AND variation (e.g. standard deviation) or associated estimates of uncertainty (e.g. confidence intervals)
☒	☐	For null hypothesis testing, the test statistic (e.g. *F*, *t*, *r*) with confidence intervals, effect sizes, degrees of freedom and *P* value noted<br>*Give P values as exact values whenever suitable.*
☒	☐	For Bayesian analysis, information on the choice of priors and Markov chain Monte Carlo settings
☒	☐	For hierarchical and complex designs, identification of the appropriate level for tests and full reporting of outcomes
☒	☐	Estimates of effect sizes (e.g. Cohen's *d*, Pearson's *r*), indicating how they were calculated

Our web collection on statistics for biologists contains articles on many of the points above.

Software and code

Policy information about availability of computer code

Data collection	No custom algorithms or software was used
Data analysis	No custom algorithms or software was used

For manuscripts utilizing custom algorithms or software that are central to the research but not yet described in published literature, software must be made available to editors/reviewers. We strongly encourage code deposition in a community repository (e.g. GitHub). See the Nature Research guidelines for submitting code & software for further information.

Data

Policy information about availability of data

All manuscripts must include a data availability statement. This statement should provide the following information, where applicable:

- Accession codes, unique identifiers, or web links for publicly available datasets
- A list of figures that have associated raw data
- A description of any restrictions on data availability

The X-ray crystallographic coordinates and structure factor files for MFN2IM structures have been deposited in the Protein Data Bank (PDB) under the following accession numbers: 6JFL for nucleotide-free MFN2IM, 6JFK for GDP-bound MFN2IM, and 6JFM for MFN2IM(T111D). The data that support the findings of this study are available from the corresponding author upon reasonable request.

Field-specific reporting

Please select the one below that is the best fit for your research. If you are not sure, read the appropriate sections before making your selection.

Life sciences study design

All studies must disclose on these points even when the disclosure is negative.

Sample size	Experiments were performed in biological triplicates and the data showed satisfying consistency
Data exclusions	No data were excluded from the analyses
Replication	All attempts at replication were successful
Randomization	Randomization is not relevant to this study because sample identities were known (wild-type or mutants) during scoring
Blinding	Blinding is not relevant to this study because sample identities were known (wild-type or mutants) during scoring

Reporting for specific materials, systems and methods

We require information from authors about some types of materials, experimental systems and methods used in many studies. Here, indicate whether each material, system or method listed is relevant to your study. If you are not sure if a list item applies to your research, read the appropriate section before selecting a response.

Materials & experimental systems

n/a	Involved in the study	
☐	☒	Antibodies
☐	☒	Eukaryotic cell lines
☒	☐	Palaeontology
☒	☐	Animals and other organisms
☒	☐	Human research participants
☒	☐	Clinical data

Methods

n/a	Involved in the study	
☒	☐	ChIP-seq
☒	☐	Flow cytometry
☒	☐	MRI-based neuroimaging

Antibodies

Antibodies used	9E10 monoclonal antibody against human c-Myc was purchased from DHSB at the University of Iowa. Host species: mouse; Myeloma Strain: SP2; Uniprot ID: P01106; Entrez Gene ID: 4609; Antibody Registry ID: AB_2266850
Validation	To validate the specificity of the 9E10 antibody, cell lines without transduction or transduced with a non-Myc-containing vector were stained. Details of the 9E10 antibody can be found at http://dshb.biology.uiowa.edu/9E-10_2/

Eukaryotic cell lines

Policy information about cell lines

Cell line source(s)	The Mfn1/Mfn2 double knock-out MEF cell line was generated in-house
Authentication	The cell line has been authenticated by genotyping with PCR
Mycoplasma contamination	The cell line is free of mycoplasma
Commonly misidentified lines (See ICLAC register)	No commonly misidentified cell lines were used
